# Three-Way Decision Models Based on Ideal Relations in Multi-Attribute Decision-Making

**DOI:** 10.3390/e24070986

**Published:** 2022-07-17

**Authors:** Xiaozhi Chen, Ligeng Zou

**Affiliations:** 1 Hunan Provincial Laboratory of Intelligent Computing and Language Information Processing, Hunan Normal University, Changsha 410081, China; ccchen@hunnu.edu.cn; 2 College of Information Science and Engineering, Hunan Normal University, Changsha 410081, China

**Keywords:** three-way decision, multi-attribute decision-making, ideal relations, decision-theoretic rough set

## Abstract

In recent years, research on applications of three-way decision (e.g., TWD) has attracted the attention of many scholars. In this paper, we combine TWD with multi-attribute decision-making (MADM). First, we utilize the essential idea of TOPSIS in MADM theory to propose a pair of new ideal relation models based on TWD, namely, the three-way ideal superiority model and the three-way ideal inferiority model. Second, in order to reduce errors caused by the subjectivity of decision-makers, we develop two new methods to calculate the state sets for the two proposed ideal relation models. Third, we employ aggregate relative loss functions to calculate the thresholds of each object, divide all objects into three different territories and sort all objects. Then, we use a concrete example of building appearance selection to verify the rationality and feasibility of our proposed models. Furthermore, we apply comparative analysis, Spearman’s rank correlation analysis and experiment analysis to illustrate the consistency and superiority of our methods.

## 1. Introduction

MADM, also known as finite scheme multi-criteria decision-making, refers to the decision problem of choosing the optimal alternative or ranking the scheme under the condition of multi-attribute. MADM is an important feature of human cognition and problem solving and plays a vital role in modern decision science. It has been widely applied in many fields such as engineering, technology, economics, management, military affairs and so forth.

In the past, decision-makers used to approach a decision-making problem based on two kinds of decisions: acceptance or rejection. However, this approach usually does not yield the optimal decisions or desired decision results. In view of this, Yao [1] put forward the concept of TWD in 2009. TWD is a decision model that is summarized and refined in the process of long-term research on rough sets, especially probabilistic rough sets and decision-theoretic rough sets, and is in line with the actual cognitive ability of human beings. TWD usually utilizes the probabilistic rough set model with two parameters, α and β, to divide the entire universe into three disjointed territories, namely positive territory, boundary territory and negative territory, and then adopts different strategies and methods for each of the three territories. In this paper, we construct a pair of new ideal relations by using the most essential ideas of TOPSIS [2], the ideal superiority relation and the ideal inferiority relation, in MADM theory [3]. Using the ideal superiority relation, we construct a TWD model based on the ideal superiority class. Similarly, the ideal inferiority class is constructed upon the ideal inferiority relation, upon which the other TWD model is proposed. Subsequently, we use the proposed models to analyze and evaluate an example of an architectural-appearance selection problem.

There are three main motivations of this paper:

(1) First of all, traditional MADM methods are generally combined with the two-way decision model, while we combine MADM with TWD. In this paper, we use the basis behind TOPSIS together with TWD in MADM. The main idea of TOPSIS is that the optimal object should have the minimal distance to the best target solution (BTS); at the same time, the larger the distance is from the worst target solution (WTS), the better. However, its limitation is that we cannot determine the order of the objects when they only meet one of the conditions or neither of the two conditions. To solve the problem, some scholars proposed equivalence relations [4], similar relations [5,6,7], dominance relations [8,9] and neighborhood operators [10,11], while we propose a new pair of ideal relations.
(2) Secondly, in most probability rough sets, the values of α and β are given artificially, and they do not answer why they should be set like this. Moreover, regarding the calculation of conditional probability [12] in decision-theoretic rough set models [1,5,13], scholars have different understanding and calculation methods from different angles and analysis directions; the properties of state sets generally have two types: classic sets [14,15] and fuzzy sets [16]. In classic sets, object membership values are given subjectively, while few studies have calculated state set values. However, different decision-makers have different opinions and preferences, and the given membership values also have great differences. Therefore, in order to reduce the error caused by subjectivity, we propose a new method of objectively calculating the state.
(3) Thirdly, since there are two states and three behaviors for each object, an object has six loss functions, each of which is either a subjective loss function [17,18,19] or an objective loss function [7,16,20]. If multiple attributes of an object are considered separately, there are six loss functions for each attribute, which requires a huge amount of calculation and lots of stored data. In this paper, we use the relative loss function proposed by Jia and Liu, which aggregates the relative loss function values for each object to reduce the amount of calculation. Besides, in order to improve the accuracy and reliability of TWD division, we calculate the threshold of each object and then divide all objects into three territories according to the threshold of each object.

The research contributions of this paper are as follows:

(1) We combine MADM with TWD and use TOPSIS to propose a pair of new ideal relations, namely ideal superiority relations and ideal inferiority relations, which have opposite definition conditions. Based on these two relations, we construct the ideal superiority class on the basis of the ideal superiority relations and the ideal inferiority class on the basis of the ideal inferiority relations. Furthermore, we construct a pair of new models: one is the TWD model based on the ideal superiority class, that is, the TWD ideal superiority model; the other is the TWD model based on the ideal inferiority class, that is, the TWD ideal inferiority model. These two models combined with TWD can be applied to the classification and sorting of objects. Moreover, the models we propose provide a new theoretical basis for research on uncertain decision-making, decision-making model selection, dynamic monitoring and intelligent decision-making technology. Meanwhile, these two models also provide new insights and ideas for decision-makers who are studying TWD.
(2) In the current paper, we provide a new calculation method for the state set of conditional probability. In Wang’s method [21], fixed values of parameters are given subjectively; however, for different decision-makers, the research limits are different, so this method has certain limitations and inflexibility. On the basis of Wang’s method, we set up an adjustable preference parameter *k* to control the cardinality of the object class, which could help calculate the values of state sets objectively to provide decision-makers with various choices. Our proposed method of calculating state sets provides new insight into the field of decision analysis.
(3) In terms of the loss function, the relative loss function of Jia and Liu is calculated objectively using the evaluation value in the information matrix. In this paper, different from the calculation methods of Jia and Liu, we set the risk-aversion coefficient of each attribute in the relative loss function to the same value instead of subjectively measuring the risk-aversion coefficient of each attribute by the decision maker, and it has been further expanded by computing the threshold of each object. The threshold is used to determine the three territories of TWD. Due to the inconsistency between the nature of attributes and the standards of the criteria for each object, we calculate the threshold of each object instead of using the same threshold standard. Hence, the measurement scale is more in line with human cognition and persuasive, and the research results obtained are more accurate and reasonable.

The specific structure of this paper is as follows: Section 2 introduces some fundamental knowledge. In Section 3, we construct a pair of new TWD models based on the ideal relations. In Section 4, we explore an application of the proposed TWD-MADM approach. In  Section 5, we conduct experimental analysis and Spearman’s correlation coefficient analysis. In Section 6, we give a brief overview of this paper and the outlook for future research.

## 2. Preliminaries

In this section, we introduce some fundamental knowledge of MADM, decision-theoretic rough sets and relative loss functions.

### 2.1. MADM

An MADM problem is about finding an optimal object from a set of related alternatives according to the specified preferences, given the attributes for each of the alternatives. In this paper, a nonempty finite set of objects is denoted by $O=\left\{O_{i}|i\in \eta \right\}\left(\eta =\left\{1,2,\cdots ,n\right\}\right)$, where $O_{i}$ is the *i*-th object. A nonempty finite set of attributes is expressed by $S=\left\{S_{j}|j\in \mu \right\}\left(\mu =\left\{1,2,\cdots ,m\right\}\right)$, where $S_{j}$ is the *j*-th attribute. Then, the pair (O,S) is called an information system. The value of object $O_{i}$ with respect to attribute $S_{j}$ is denoted by $S_{j}\left(O_{i}\right)$ (i.e., $u_{ij}$). If there exists $u_{ij}\in S$ and $u_{ij}$ is a fuzzy attribute, i.e., $u_{ij}\in \left(0,1\right)$, then (O,S) is referred to as a fuzzy information system. If each attribute of *S* is fuzzy, then (O,S) is called a full fuzzy information system. Here, a fuzzy information system is represented by I=(O,S), and $W=\{w_{j}|j\in \mu \}$ is the attribute weight vector set, where $w_{j}$ shall satisfy two conditions: $0\leq w_{j}\leq 1$ and $\sum_{j=1}^{m}w_{j}=1$. For the sake of simplicity, all information systems in this paper refer to fuzzy information systems unless specifically stated. A fuzzy information system can be illustrated as an n×m MADM information matrix. Usually, we choose an optimal alternative from *O* by evaluating and ranking all objects under the *m* attributes. Regarding the above description, in what follows, Table 1 demonstrates the multi-attribute information matrix.

In Table 1, there can be multiple types of attributes, such as profit attributes, expense attributes, public attributes, private attributes and so on. Accordingly, in order to classify and sort all objects accurately as well as to obtain the expected ranking result, we need to unify these diverse attributes into the same dimension before making a decision, which requires a normalized decision-making matrix. The normalized decision-making matrix is shown in Table 2. For any decision-making problem, using the same dimension and standard to measure different objects makes the decision-making process easier and simpler, and the decision-making results are more convincing.

### 2.2. The TWD Model Based on Decision-Theoretic Rough Set (DTRS)

The decision-theoretic rough set model is based on the process of the Bayesian decision and the main idea of TWD. The decision-theoretic rough set model uses two states and three actions to describe the decision-making process. In this paper, we let Ω={R,¬R} be a set of states, which means an object is either in R or not in R. Meanwhile, let $T=\{T_{P},T_{B},T_{N}\}$ be a set of actions, where $T_{P},T_{B},T_{N}$ are used to classify an object into three categories. When object $O_{i}\in R$, we classify $O_{i}$ based on which one of the following conditions holds true: $O_{i}\in POS\left(R\right)$, $O_{i}\in BND\left(R\right)$ or $O_{i}\in NEG\left(R\right)$.

Considering that different actions will result in diverse losses correspondingly, we respectively let $\theta_{i}^{PP}$, $\theta_{i}^{BP}$ and $\theta_{i}^{NP}$ represent the loss functions of selecting the action $T_{P}$, $T_{B}$ and $T_{N}$ when the object $O_{i}$ belongs to *R*. Likewise, when $O_{i}\in \neg R$, $\theta_{i}^{PN}$, $\theta_{i}^{BN}$ and $\theta_{i}^{NN}$ represent the loss functions for choosing the action $T_{P}$, $T_{B}$ and $T_{N}$, respectively. At the same time, let $\left[O_{i}\right]$ stand for the ideal class of $O_{i}$ that we construct in this paper.

For an object $O_{i}$, the expected losses $Q\left(T⋄|\left[O_{i}\right]\right)\left(⋄=P,B,N\right)$ of choosing three different actions can be calculated by the following formulas:

(1) $$Q\left(T_{P}|\left[O_{i}\right]\right)=\theta_{i}^{PP}P\left(R|\left[O_{i}\right]\right)+\theta_{i}^{PN}P\left(\neg R|\left[O_{i}\right]\right),$$

(2) $$Q\left(T_{B}|\left[O_{i}\right]\right)=\theta_{i}^{BP}P\left(R|\left[O_{i}\right]\right)+\theta_{i}^{BN}P\left(\neg R|\left[O_{i}\right]\right),$$

(3) $$Q\left(T_{N}|\left[O_{i}\right]\right)=\theta_{i}^{NP}P\left(R|\left[O_{i}\right]\right)+\theta_{i}^{NN}P\left(\neg R|\left[O_{i}\right]\right),$$

where $P(R|\left[O_{i}\right])$ is the conditional probability of an alternative $O_{i}$ when it is in *R*. In the same way, $P(\neg R|\left[O_{i}\right])$ represents the conditional probability of an alternative $O_{i}$ when it’s not in *R*.

According to the Bayesian minimal risk decision theory, the set of actions with the least expected loss is chosen as the remarkable course of action. Hence, we can draw the corresponding three decision rules as follows:

$$\begin{array}{cc} \left(P\right)\;\mathrm{If}\; & Q\left(T_{P}|\left[O_{i}\right]\right)\leq Q\left(T_{B}|\left[O_{i}\right]\right)\;\mathrm{and}\;Q\left(T_{P}|\left[O_{i}\right]\right)\leq Q\left(T_{N}|\left[O_{i}\right]\right),\;\mathrm{then}\;O_{i}\in POS\left(R\right),\\ \left(B\right)\;\mathrm{If}\; & Q\left(T_{B}|\left[O_{i}\right]\right)\leq Q\left(T_{P}|\left[O_{i}\right]\right)\;\mathrm{and}\;Q\left(T_{B}|\left[O_{i}\right]\right)\leq Q\left(T_{N}|\left[O_{i}\right]\right),\;\mathrm{then}\;O_{i}\in BND\left(R\right),\\ \left(N\right)\;\mathrm{If}\; & Q\left(T_{N}|\left[O_{i}\right]\right)\leq Q\left(T_{P}|\left[O_{i}\right]\right)\;\mathrm{and}\;Q\left(T_{N}|\left[O_{i}\right]\right)\leq Q\left(T_{B}|\left[O_{i}\right]\right),\;\mathrm{then}\;O_{i}\in NEG\left(R\right). \end{array}$$

In light of the properties of the probability function, we conclude that $P(R|\left[O_{i}\right])$ + $P(\neg R|\left[O_{i}\right])$=1. From the semantic interpretation of risk in real life, we suppose that $\theta_{i}^{PP}\leq \theta_{i}^{BP}<\theta_{i}^{NP}$ and $\theta_{i}^{NN}\leq \theta_{i}^{BN}<\theta_{i}^{PN}$. Thus, the rules (P)−(N) can be simplified as follows:

$$\begin{array}{cc} \left(P1\right)\;\mathrm{If}\; & P\left(R|\left[O_{i}\right]\right)\geq \alpha_{i}\;\mathrm{and}\;P\left(R|\left[O_{i}\right]\right)\geq \gamma_{i},\mathrm{then}\;O_{i}\in POS\left(R\right),\\ \left(B1\right)\;\mathrm{If}\; & P\left(R|\left[O_{i}\right]\right)\leq \alpha_{i}\;\mathrm{and}\;P\left(R|\left[O_{i}\right]\right)\geq \beta_{i},\mathrm{then}\;O_{i}\in BND\left(R\right),\\ \left(N1\right)\;\mathrm{If}\; & P\left(R|\left[O_{i}\right]\right)\leq \beta_{i}\;\mathrm{and}\;P\left(R|\left[O_{i}\right]\right)\leq \gamma_{i},\mathrm{then}\;O_{i}\in NEG\left(R\right), \end{array}$$

where

(4) $$\alpha_{i}=\frac{\theta_{i}^{PN}-\theta_{i}^{BN}}{\left(\theta_{i}^{PN}-\theta_{i}^{BN}\right)+\left(\theta_{i}^{BP}-\theta_{i}^{PP}\right)},$$

(5) $$\beta_{i}=\frac{\theta_{i}^{BN}-\theta_{i}^{NN}}{\left(\theta_{i}^{BN}-\theta_{i}^{NN}\right)+\left(\theta_{i}^{NP}-\theta_{i}^{BP}\right)},$$

(6) $$\gamma_{i}=\frac{\theta_{i}^{PN}-\theta_{i}^{NN}}{\left(\theta_{i}^{PN}-\theta_{i}^{NN}\right)+\left(\theta_{i}^{NP}-\theta_{i}^{NN}\right)}.$$

To find out the magnitude relationship between the three thresholds α,β and γ, we have two reasonable assumptions based on the magnitude of the loss functions:

Given $\left(\theta_{i}^{PN}-\theta_{i}^{BN}\right)\left(\theta_{i}^{NP}-\theta_{i}^{BP}\right)>\left(\theta_{i}^{BP}-\theta_{i}^{PP}\right)\left(\theta_{i}^{BN}-\theta_{i}^{NN}\right)$, we have $0\leq \beta_{i}<\gamma_{i}<\alpha_{i}\leq 1$. As a result, the above decision rules of (P1)–(N1) can be further simplified as follows:

$$\begin{array}{cc} \left(P2\right)\;\mathrm{If}\; & P\left(R|\left[O_{i}\right]\right)\geq \alpha_{i},\;\mathrm{then}\;O_{i}\in POS\left(R\right),\\ \left(B2\right)\;\mathrm{If}\; & \beta_{i}<P\left(R|\left[O_{i}\right]\right)<\alpha_{i},\;\mathrm{then}\;O_{i}\in BND\left(R\right),\\ \left(N2\right)\;\mathrm{If}\; & P\left(R|\left[O_{i}\right]\right)\leq \beta_{i},\;\mathrm{then}\;O_{i}\in NEG\left(R\right). \end{array}$$

Given $\left(\theta_{i}^{PN}-\theta_{i}^{BN}\right)\left(\theta_{i}^{NP}-\theta_{i}^{BP}\right)\leq \left(\theta_{i}^{BP}-\theta_{i}^{PP}\right)\left(\theta_{i}^{BN}-\theta_{i}^{NN}\right)$, we have $0<\alpha_{i}\leq \gamma_{i}\leq \beta_{i}<1$. As a result, the decision rules of (P1)–(N1) above can be further simplified as follows:

$$\begin{array}{cc} \left(P3\right)\;\mathrm{If}\; & P\left(R|\left[O_{i}\right]\right)\geq \gamma_{i},\;\mathrm{then}\;O_{i}\in POS\left(R\right),\\ \left(N3\right)\;\mathrm{If}\; & P\left(R|\left[O_{i}\right]\right)<\gamma_{i},\;\mathrm{then}\;O_{i}\in NEG\left(R\right). \end{array}$$

### 2.3. The Relative Loss Functions

To reduce the computational cost of thresholds, Jia and Liu [3] recently rewrote the calculation formulas of the three thresholds:

(7) $$\alpha =\frac{\theta^{(P-B)N}}{\theta^{(P-B)N}+\theta^{(B-P)P}},$$

(8) $$\beta =\frac{\theta^{(B-N)N}}{\theta^{(B-N)N}+\theta^{(N-B)P}},$$

(9) $$\gamma =\frac{\theta^{(P-N)N}}{\theta^{(P-N)N}+\theta^{(N-P)P}},$$

where $\theta^{(N-P)P}$ represents the difference between the boundary territory and the positive territory when an object belongs to *R*. Other loss functions have similar explanatory principles.

The relative loss functions simplify the calculation process of the original loss functions, i.e., the loss is zero when accepting the action is correct or rejecting the action is wrong. Jia and Liu also carried out a regular transformation on loss functions, i.e., the loss functions in *R*: $\theta_{\prime }^{PP}=0,\;\theta_{\prime }^{BP}=\theta^{BP}-\theta^{PP},\;\theta_{\prime }^{NP}=\theta^{NP}-\theta^{PP}$; the loss functions in ¬R: $\theta_{\prime }^{PN}=\theta^{PN}-\theta^{NN},\;\theta_{\prime }^{BN}=\theta^{BN}-\theta^{NN},\;\theta_{\prime }^{NN}=0$. The results are shown in Table 3.

For example, the relative loss functions of object $O_{i}$ under attribute $S_{j}$ is shown in Table 4.

In Table 4, σ stands for the risk-aversion coefficient, whose value range has certain requirements. In this paper we focus on TWD, therefore σ∈[0,0.5]. Furthermore, in an MADM decision problem, there is more than one attribute that needs to be considered for an object. If the loss functions need to be calculated for each attribute, the whole process will be cumbersome and time-consuming. Hence, we aggregate the relative loss function values of each object to reduce the computational cost. The results are shown in Table 5.

## 3. TWD Models Based on the Ideal Relations

In this section, we construct a pair of ideal relations with opposite definitions by applying the TOPSIS method. Then we explore the ideal relations to construct two TWD ideal models. In this section, $O=\{O_{i}|i\in \eta \}$(η={1,2,⋯,n}) is the object set.

### 3.1. A TWD Model Based on the Ideal Superiority Relation

In the following, we introduce in detail how to construct the ideal superiority relation and the ideal superiority class, as well as relevant definitions and theorems. Then, we describe the process of establishing the TWD model with DTRS by using our proposed superiority class.

#### 3.1.1. Construction of the Ideal Superiority Relation and Class

According to the TOPSIS method, for any two objects $O_{i}$ and $O_{j}$, if the distance between $O_{i}$ and the BTS is less than the distance between $O_{j}$ and the BTS, and the distance between $O_{i}$ and the WTS is greater than the distance between $O_{j}$ and the WTS, then we can conclude that $O_{i}$ precedes $O_{j}$.

In what follows, based on Table 2, we name the distance between $O_{i}$ and the BTS as the best target ideal distance (BTID), represented by $O_{i}^{+}$. In the same way, the distance between $O_{i}$ and the WTS is referred to as the worst target ideal distance (WTID), denoted by $O_{i}^{-}$. According to the above definitions, $O_{i}^{+}$ and $O_{i}^{-}$ can be computed as follows.

(10) $$O_{i}^{+}=\sqrt{\sum_{j=1}^{m}w_{j}{\left(v_{ij}-\max_{i\in \eta }v_{ij}\right)}^{2}},O_{i}^{-}=\sqrt{\sum_{j=1}^{m}w_{j}{\left(v_{ij}-\min_{i\in \eta }v_{ij}\right)}^{2}}.$$

Utilizing $O_{i}^{+}$ and $O_{i}^{-}$, the ideal superiority relation based on the TOPSIS method is described below.

**Definition** **1.** *Based on the fuzzy information system I=(O,S), e.g., Table 2, we define the ideal superiority relation as follows:*

(11) $$E=\{\left(O_{i},O_{j}\right)\in O\times O|O_{j}^{+}\leq O_{i}^{+}\;\mathrm{and}\;O_{j}^{-}\geq O_{i}^{-},j\in \eta \}.$$

**Remark** **1.** *The explanation for Formula (11): if the BTID of $O_{j}$ is less than or equal to that of $O_{i}$, and, simultaneously, the WTID of $O_{j}$ is greater than or equal to that of $O_{i}$, then $(O_{i},O_{j})\in E$, i.e., $O_{j}$ superior to $O_{i}$. From the perspective of profit and expense attributes, the lower the expense, the higher the profit, which is the optimal decision effect.*

**Definition** **2.** *Based on fuzzy information system I=(O,S) and Definition 1, the ideal superiority class of $O_{i}$ is constructed as follows:*

(12) $${\left[O_{i}\right]}_{E}=\left\{O_{j}|O_{j}^{+}\leq O_{i}^{+}\;\mathrm{and}\;O_{j}^{-}\geq O_{i}^{-},O_{j}\in O\right\}.$$

Obviously, the ideal superiority class of object $O_{i}$ is a collection of objects that are superior to $O_{i}$.

**Example** **1.** *Let us take a simple fund selection problem as an example to explain the above definition. There are five available funds $S=\{s_{1},s_{2},s_{3},s_{4},s_{5}\}$ and four related attributes $R=\{r_{1},r_{2},r_{3},r_{4}\}$. Among them, $r_{1}$ and $r_{3}$ are profit attributes, while $r_{2}$ and $r_{4}$ are expense attributes; w={0.3,0.1,0.2,0.4} is the weight vector of these four attributes; $\tau =\{\tau_{1},\tau_{2},\tau_{3},\tau_{4}\}$ is the risk avoidance coefficient of these four attributes. The specific values are presented in Table 6:*

According to the steps of the TOPSIS model and Definition 2, we can calculate the ideal superiority classes of these five funds as follows:

${\left[s_{1}\right]}_{E}=\left\{s_{1},s_{3},s_{4}\right\}$.

${\left[s_{2}\right]}_{E}=\left\{s_{2},s_{3},s_{4},s_{5}\right\}$.

${\left[s_{3}\right]}_{E}=\left\{s_{3}\right\}$.

${\left[s_{4}\right]}_{E}=\left\{s_{3},s_{4}\right\}$.

${\left[s_{5}\right]}_{E}=\left\{s_{3},s_{4},s_{5}\right\}$.

**Proposition** **1.** *For the ideal superiority relation E, we summarize that it has the following properties:*

*(a) Reflexivity: For $O_{i}\in O\;\left(i\in \eta \right)$, if it satisfies $O_{i}\in {\left[O_{i}\right]}_{E}$, then ${\left[O_{i}\right]}_{E}$ possesses reflexivity;*

*(b) Transitivity: For $O_{x},O_{y},O_{z}\in O\;\left(x,y,z\in \eta \right)$, if $O_{x}\in {\left[O_{y}\right]}_{E}$ and $O_{y}\in {\left[O_{z}\right]}_{E}$, then we can draw that $O_{x}\in {\left[O_{z}\right]}_{E}$.*

**Proof.**  As can be seen from Definition 2, the above two properties are easily proven.    □

**Definition** **3.** *In light of Definition 1, we construct a brand new state set Π={T1,T2}, where T1 represents a collection of outstanding objects, and T2 represents a collection of non-outstanding objects. The state set of the ideal superiority class is defined as follows:*

$$\begin{array}{c} T1=\left\{O_{i}|\frac{|{\left[O_{i}\right]}_{E}|}{\left|n\right|}\leq k_{1}\right\},\;T2=\left\{O_{i}|\frac{|{\left[O_{i}\right]}_{E}|}{\left|n\right|}>k_{1}\right\},k_{1}\in \left(0,0.5\right], \end{array}$$

where $|{\left[O_{i}\right]}_{E}|$ is the cardinality of the ideal superiority class of $O_{i}$, and $k_{1}$ is called the preference parameter of the ideal superiority class. When the two states are constructed, the conditional probability under the ideal superiority class $P(*|{\left[O_{i}\right]}_{E})$ can be computed as $P\left(*|{\left[O_{i}\right]}_{E}\right)=\frac{|*⋂{\left[O_{i}\right]}_{E}|}{|{\left[O_{i}\right]}_{E}|}\;\left(*=\left\{T1,T2\right\}\right)$.

The selection of $k_{1}$ is based on the decision-maker’s preference, the perspective of the problem or the research direction. Every decision-maker has different standards for the measurement of problems. For example, some decision-makers require the definition of excellence to be extremely strict, i.e., all conditions must be met. On the other hand, for some decision-makers the standard is loosened and only a certain number of conditions need to be met. In view of the above-mentioned reasons, different researchers will select different preference parameters for the same decision problem, and the resulting decisions will be diverse as well.

For preference parameter $k_{1}$ of the ideal superiority class, we set it between 0 and 0.5, and the lower limit is 0, and it must be an open interval. If it is a closed interval, the decision conditions are too harsh, resulting in all objects belonging to a state set. The upper limit of 0.5 is considered to determine excellence based on the principle of majority. For example, if the number of the ideal superiority classes of object $O_{i}$ is greater than half of the object set, it indicates that object $O_{i}$ is a non-outstanding object.

For any object $O_{i}$, if the cardinality of the ideal superiority class of $O_{i}$ divided by the total number of objects is less than or equal to $k_{1}$, we say that $O_{i}$ belongs to the outstanding objects; the reason for taking the equal sign here is that when we define the ideal superiority class, we include the object itself to avoid taking an empty set. On the contrary, if the cardinality of the ideal superiority class of $O_{i}$ divided by the total number of objects is greater than $k_{1}$, then $O_{i}$ belongs to non-outstanding objects. Obviously, for the states T1 and T2, when the preference parameter $k_{1}$ grows larger, the number of objects in $T_{1}$ becomes bigger, while the number of objects in $T_{2}$ becomes smaller. On the contrary, when $k_{1}$ decreases, the cardinality of $T_{1}$ decreases, whereas the cardinality of $T_{2}$ increases.

**Remark** **2.** *The state set of the ideal superiority class is Π={T1,T2}. It possesses the following two properties: (1) T1⋃T2=O, (2) T1⋂T2=ϕ.*

From the above analysis and discussion, the union of the two states is the set of all objects, namely, the object set *O*; it indicates that all objects are assigned to one of two states. At the same time, the intersection of two form sets is an empty set, it means that it is impossible for an object to belong to both state sets in the meantime.

**Example** **2.** *(Continued from Example 1) Suppose that $k_{1}=0.3$. In line with Definition 3, we can obtain the state set of the ideal superiority class as follows:*

$$\begin{array}{c} T1=\left\{s_{3}\right\},\;T2=\left\{s_{1},s_{2},s_{4},s_{5}\right\}. \end{array}$$

Knowing the ideal superiority class set and state set of each object, we next calculate the conditional probability of each object in different states under the ideal superiority class.

$P\left(T1|{\left[s_{1}\right]}_{E}\right)=\frac{|T1⋂{\left[s_{1}\right]}_{E}|}{|{\left[s_{1}\right]}_{E}|}=\frac{|\left\{s_{3}\right\}⋂\left\{s_{1},s_{3},s_{4}\right\}|}{\left|\{s_{1},s_{3},s_{4}\}\right|}=\frac{1}{3}$.

$P\left(T1|{\left[s_{2}\right]}_{E}\right)=\frac{|T1⋂{\left[s_{2}\right]}_{E}|}{|{\left[s_{2}\right]}_{E}|}=\frac{|\left\{s_{3}\right\}⋂\left\{s_{2},s_{3},s_{4},s_{5}\right\}|}{\left|\{s_{2},s_{3},s_{4},s_{5}\}\right|}=\frac{1}{4}$.

$P\left(T1|{\left[s_{3}\right]}_{E}\right)=\frac{|T1⋂{\left[s_{3}\right]}_{E}|}{|{\left[s_{3}\right]}_{E}|}=\frac{|\left\{s_{3}\right\}⋂\left\{s_{3}\right\}|}{\left|\{s_{3}\}\right|}=1$.

$P\left(T1|{\left[s_{4}\right]}_{E}\right)=\frac{|T1⋂{\left[s_{4}\right]}_{E}|}{|{\left[s_{4}\right]}_{E}|}=\frac{|\left\{s_{3}\right\}⋂\left\{s_{3},s_{4}\right\}|}{\left|\{s_{3},s_{4}\}\right|}=\frac{1}{2}$.

$P\left(T1|{\left[s_{5}\right]}_{E}\right)=\frac{|T1⋂{\left[s_{5}\right]}_{E}|}{|{\left[s_{5}\right]}_{E}|}=\frac{|\left\{s_{3}\right\}⋂\left\{s_{3},s_{4},s_{5}\right\}|}{\left|\{s_{3},s_{4},s_{5}\}\right|}=\frac{1}{3}$.

   In the same way, we can replace the T1 state with T2 and perform the same calculation method to obtain the conditional probability of each ideal superiority class object in the T2 state.

#### 3.1.2. The Three-Way Process Based on the Ideal Superiority Class

In the decision-theoretic rough set, each decision behavior has a corresponding risk loss, which means various actions will produce different loss functions. Since each object has two possible states and three possible actions in DTRS, there are six loss functions in total for each object. In the proposed ideal superiority class and state set, the symbols $L_{P},L_{B},L_{N}$ express acceptance, deferred consideration and rejection, respectively. Subsequently, acceptance, deferred consideration and rejection represent the positive, boundary and negative territory in TWD, respectively.

In this paper, we use the relative loss functions proposed by Jia and Liu [3]. When an object belongs to T1, taking the loss of action $L_{P}$ as the standard, $\theta_{{\left[O_{i}\right]}_{E}}^{PT1}$, $\theta_{{\left[O_{i}\right]}_{E}}^{BT1}$ and $\theta_{{\left[O_{i}\right]}_{E}}^{NT1}$ minus $\theta_{{\left[O_{i}\right]}_{E}}^{PT1}$ respectively; then the relative losses of $L_{P},L_{B}$ and $L_{N}$ are 0, $\theta_{{\left[O_{i}\right]}_{E}}^{{\left(BT1\right)}^{*}}$ and $\theta_{{\left[O_{i}\right]}_{E}}^{{\left(NT1\right)}^{*}}$, respectively; when an object belongs to T2, taking the loss of action $L_{N}$ as the standard, $\theta_{{\left[O_{i}\right]}_{E}}^{PT2}$, $\theta_{{\left[O_{i}\right]}_{E}}^{BT2}$ and $\theta_{{\left[O_{i}\right]}_{E}}^{NT2}$ minus $\theta_{{\left[O_{i}\right]}_{E}}^{NT2}$, respectively, then the relative losses of $L_{P},L_{B}$ and $L_{N}$ are $\theta_{{\left[O_{i}\right]}_{E}}^{{\left(PT2\right)}^{*}}$, $\theta_{{\left[O_{i}\right]}_{E}}^{{\left(BT2\right)}^{*}}$ and 0, respectively.

For an object $O_{i}$ in T1, the risk of dividing it into the positive territory is less than or equal to the risk of dividing it into the boundary territory; both risks are smaller than the risk of dividing it into the negative territory. Similarly, for an object $O_{i}$ in T2, the risk of dividing it into the negative territory is less than or equal to the risk of dividing it into the boundary territory; both risks are smaller than the risk of dividing it into the positive territory. Therefore, we put forward a reasonable hypothesis with practical significance:

$0\leq \theta_{{\left[O_{i}\right]}_{E}}^{{\left(BT1\right)}^{*}}<\theta_{{\left[O_{i}\right]}_{E}}^{{\left(NT1\right)}^{*}}$,

$0\leq \theta_{{\left[O_{i}\right]}_{E}}^{{\left(BT2\right)}^{*}}<\theta_{{\left[O_{i}\right]}_{E}}^{{\left(PT2\right)}^{*}}$.

Based on Definitions 2 and 3, as well as the expected risk formulas, the expected losses when $O_{i}$ takes actions are:

(13) $$Q\left(L_{P}|{\left[O_{i}\right]}_{E}\right)=\theta_{{\left[O_{i}\right]}_{E}}^{{\left(PT2\right)}^{*}}P\left(T2|{\left[O_{i}\right]}_{E}\right),$$

(14) $$Q\left(L_{B}|{\left[O_{i}\right]}_{E}\right)=\theta_{{\left[O_{i}\right]}_{E}}^{{\left(BT1\right)}^{*}}P\left(T1|{\left[O_{i}\right]}_{E}\right)+\theta_{{\left[O_{i}\right]}_{E}}^{{\left(BT2\right)}^{*}}P\left(T2|{\left[O_{i}\right]}_{E}\right),$$

(15) $$Q\left(L_{N}|{\left[O_{i}\right]}_{E}\right)=\theta_{{\left[O_{i}\right]}_{E}}^{{\left(NT1\right)}^{*}}P\left(T1|{\left[O_{i}\right]}_{E}\right).$$

The property that $P\left(T1|{\left[O_{i}\right]}_{E}\right)+P\left(T2|{\left[O_{i}\right]}_{E}\right)=1$ implies $P\left(T2|{\left[O_{i}\right]}_{E}\right)=1-P\left(T1|{\left[O_{i}\right]}_{E}\right)$. As a result, we can simplify the above formulas as follows:

(16) $$Q\left(L_{P}|{\left[O_{i}\right]}_{E}\right)=\theta_{{\left[O_{i}\right]}_{E}}^{{\left(PT2\right)}^{*}}\left(1-P\left(T1|{\left[O_{i}\right]}_{E}\right)\right),$$

(17) $$Q\left(L_{B}|{\left[O_{i}\right]}_{E}\right)=\theta_{{\left[O_{i}\right]}_{E}}^{{\left(BT1\right)}^{*}}P\left(T1|{\left[O_{i}\right]}_{E}\right)+\theta_{{\left[O_{i}\right]}_{E}}^{{\left(BT2\right)}^{*}}\left(1-P\left(T1|{\left[O_{i}\right]}_{E}\right)\right),$$

(18) $$Q\left(L_{N}|{\left[O_{i}\right]}_{E}\right)=\theta_{{\left[O_{i}\right]}_{E}}^{{\left(NT1\right)}^{*}}P\left(T1|{\left[O_{i}\right]}_{E}\right).$$

The practical implication of the Bayesian decision process, according to the minimum risk principle, is that an action is performed when the risk of the action does not exceed the risk of taking the other two choices (in the form of dividing an object into the corresponding territory). Hence, we can represent the divisions of TWD as follows:

$$\begin{array}{cc} \left(P4\right)\;\mathrm{If}\;Q\left(L_{P}|{\left[O_{i}\right]}_{E}\right)\leq Q\left(L_{B}|{\left[O_{i}\right]}_{E}\right)\;\mathrm{and}\;Q\left(L_{P}|{\left[O_{i}\right]}_{E}\right)\leq Q\left(L_{N}|{\left[O_{i}\right]}_{E}\right), & \;\mathrm{then}\;O_{i}\in POS\left(T1\right),\\ \left(B4\right)\;\mathrm{If}\;Q\left(L_{B}|{\left[O_{i}\right]}_{E}\right)\leq Q\left(L_{P}|{\left[O_{i}\right]}_{E}\right)\;\mathrm{and}\;Q\left(L_{B}|{\left[O_{i}\right]}_{E}\right)\leq Q\left(L_{N}|{\left[O_{i}\right]}_{E}\right), & \;\mathrm{then}\;O_{i}\in BND\left(T1\right),\\ \left(N4\right)\;\mathrm{If}\;Q\left(L_{N}|{\left[O_{i}\right]}_{E}\right)\leq Q\left(L_{P}|{\left[O_{i}\right]}_{E}\right)\;\mathrm{and}\;Q\left(L_{N}|{\left[O_{i}\right]}_{E}\right)\leq Q\left(L_{B}|{\left[O_{i}\right]}_{E}\right), & \;\mathrm{then}\;O_{i}\in NEG\left(T1\right). \end{array}$$

Since different attributes of an object correspond to different loss functions, we need to integrate the loss functions to reduce the amount of calculation. Table 7 displays the aggregate relative loss functions.

In Table 7, $v_{max}=\max_{j\in \mu }v_{ij}$, $v_{min}=\min_{j\in \mu }v_{ij}$, and τ∈(0,0.5) is the risk avoidance coefficient. The risk avoidance coefficient τ is determined by the decision-makers according to the characteristics of the attribute. Consequently, the values of τ for realistic problems vary.

Based on the aggregate relative loss functions exhibited in Table 7, the thresholds $\alpha_{i}^{E},\beta_{i}^{E}\mathrm{and}\;\gamma_{i}^{E}$ of $O_{i}$ can be computed as below:

(19) $$\alpha_{i}^{E}=\frac{\Sigma_{j}w_{j}\left(1-\tau \right)\left(v_{\max}-v_{ij}\right)}{\Sigma_{j}w_{j}\left(1-\tau \right)\left(v_{\max}-v_{ij}\right)+(\Sigma_{j}w_{j}\tau \left(v_{ij}-v_{\min}\right)},$$

(20) $$\beta_{i}^{E}=\frac{\Sigma_{j}w_{j}\tau \left(v_{\max}-v_{ij}\right)}{\Sigma_{j}w_{j}\tau \left(v_{\max}-v_{ij}\right)+\Sigma_{j}w_{j}\left(1-\tau \right)\left(v_{ij}-v_{\min}\right)},$$

(21) $$\gamma_{i}^{E}=\frac{\Sigma_{j}w_{j}\left(v_{\max}-v_{ij}\right)}{v_{\max}-v_{\min}}.$$

Given the three thresholds above, the rules of TWD can be rewritten as follows:

$$\begin{array}{cc} \left(P5\right)\;\mathrm{If}\; & P\left(T1|{\left[O_{i}\right]}_{E}\right)\geq \alpha_{i}^{E}\;\mathrm{and}\;P\left(T1|{\left[O_{i}\right]}_{E}\right)\geq \gamma_{i}^{E},\mathrm{then}\;O_{i}\in POS\left(T1\right),\\ \left(B5\right)\;\mathrm{If}\; & P\left(T1|{\left[O_{i}\right]}_{E}\right)\leq \alpha_{i}^{E}\;\mathrm{and}\;P\left(T1|{\left[O_{i}\right]}_{E}\right)\geq \beta_{i}^{E},\mathrm{then}\;O_{i}\in BND\left(T1\right),\\ \left(N5\right)\;\mathrm{If}\; & P\left(T1|{\left[O_{i}\right]}_{E}\right)\leq \beta_{i}^{E}\;\mathrm{and}\;P\left(T1|{\left[O_{i}\right]}_{E}\right)\leq \gamma_{i}^{E},\mathrm{then}\;O_{i}\in NEG\left(T1\right). \end{array}$$

From Section 2.3, we can see that when $0\leq \beta_{i}^{E}<\gamma_{i}^{E}<\alpha_{i}^{E}\leq 1$, the conditions of TWD are met. Therefore, we can further simplify the decision rules in (P3)−(N3) as follows:

$$\begin{array}{cc} \left(P6\right)\;\mathrm{If}\; & P\left(T1|{\left[O_{i}\right]}_{E}\right)\geq \alpha_{i}^{E},\mathrm{then}\;O_{i}\in POS\left(T1\right),\\ \left(B6\right)\;\mathrm{If}\; & \beta_{i}^{E}<P\left(T1|{\left[O_{i}\right]}_{E}\right)<\alpha_{i}^{E},\mathrm{then}\;O_{i}\in BND\left(T1\right),\\ \left(N6\right)\;\mathrm{If}\; & P\left(T1|{\left[O_{i}\right]}_{E}\right)\leq \beta_{i}^{E},\mathrm{then}\;O_{i}\in NEG\left(T1\right). \end{array}$$

**Example** **3.** *(Continued from Examples 1 and 2) In order to make the three-way process based on the ideal superiority class method easier to understand, we will explain in detail with the help of examples. Assume τ={0.4,0.4,0.4,0.4}, next we give the specific calculation process of the relative loss function and threshold of object $s_{1}$ and the classification process of TWD.*

Since we have obtained the aggregate relative loss function of object $s_{1}$ in Table 8 above, we can calculate the three thresholds of object $s_{1}$ according to Formulas (19)–(21).

$$\begin{array}{cccc} \; & \alpha_{1}^{E}=\frac{0.3581}{0.3581+0.1168}=0.7540, & \beta_{1}^{E}=\frac{0.2387}{0.2387+0.1752}=0.5767, & \gamma_{1}^{E}=\frac{0.5968}{1.0000-0.1111}=0.6714. \end{array}$$

Now that we know the $\alpha_{1}^{E},\beta_{1}^{E},\gamma_{1}^{E}$ and the conditional probability $P(T1|{\left[s_{1}\right]}_{E})$ of object $s_{1}$, according to the rules of TWD, we get $P\left(T1|{\left[s_{1}\right]}_{E}\right)=0.3333<\beta_{1}^{E}=0.5767$; then, object $s_{1}$ is divided into the negative territory. The thresholds of other objects can be obtained in the same way. In Example 2, we have obtained the conditional probability of all objects. Finally, according to the rules of (P6)–(N6), the division results of the remaining objects are presented in Table 9:

### 3.2. A TWD Model Based on the Ideal Inferiority Relation

This subsection explains in detail how to construct the ideal inferiority relation and class, as well as the essential definitions and theorems. Then, we discuss the process of developing the TWD model with DTRS by utilizing our proposed inferiority class.

#### 3.2.1. The Construction of the Ideal Inferiority Relation and Class

Similar to Section 3.1, we make use of the TOPSIS method to establish the ideal inferiority relation and the ideal inferiority class, which are defined as follows:

**Definition** **4.** *Based on the fuzzy information system I=(O,S), we define the ideal inferiority relation as follows:*

(22) $$F=\{\left(O_{i},O_{j}\right)\in O\times O|O_{j}^{+}\geq O_{i}^{+}\;\mathrm{and}\;O_{j}^{-}\leq O_{i}^{-},j\in \eta \}.$$

**Remark** **3.** *The semantic explanation of the ideal inferiority relation: Given two objects $O_{i}$ and $O_{j}$, if the BTID of $O_{j}$ is greater than or equal to that of $O_{i}$, and simultaneously the WTID of $O_{j}$ is less than or equal to that of $O_{i}$, then $(O_{i},O_{j})\in F$. In terms of profit and expense attributes, the higher the expense, the lower the profit. High expenses and low earnings are the worst types of decision-making outcomes for decision-makers.*

**Definition** **5.** *Based on the fuzzy information system I=(O,S) and Definition 4, for $\forall \;O_{i}\in O$, the ideal inferiority class of $O_{i}$ is constructed as follows:*

(23) $${\left[O_{i}\right]}_{F}=\left\{O_{j}|O_{j}^{+}\geq O_{i}^{+}\;\mathrm{and}\;O_{j}^{-}\leq O_{i}^{-},O_{j}\in O\right\}.$$

The ideal inferiority class of $O_{i}$ is the set of all objects whose BTID is greater than that of $O_{i}$, and whose WTID is smaller than that of $O_{i}$, i.e., the set of all objects inferior to object $O_{i}$.

**Example** **4.** *To illustrate the above definitions better, we use a tableware color selection problem as an example. There are six tableware colors to pick from $B=\{B_{1},B_{2},B_{3},B_{4},B_{5},B_{6}\}$, with five corresponding attributes $L=\{L_{1},L_{2},L_{3},L_{4},L_{5}\}$. Among them, $L_{2},L_{3}\;\mathrm{and}\;L_{4}$ are profit attributes, $L_{1},L_{5}$ are expense attributes, and w={0.1,0.1,0.2,0.3,0.3} are the weights of these five attributes. The multi-attribute information matrix in Table 10 represents the specific values of this project:*

According to the steps of the TOPSIS model and Definition 5, we can calculate the ideal inferiority classes of these six tableware colors as follows:

${\left[B_{1}\right]}_{F}=\left\{B_{1},B_{2},B_{3},B_{5},B_{6}\right\}$.

${\left[B_{2}\right]}_{F}=\left\{B_{2}\right\}$.

${\left[B_{3}\right]}_{F}=\left\{B_{2},B_{3}\right\}$.

${\left[B_{4}\right]}_{F}=\left\{B_{1},B_{2},B_{3},B_{4},B_{5},B_{6}\right\}$.

${\left[B_{5}\right]}_{F}=\left\{B_{2},B_{5}\right\}$.

${\left[B_{6}\right]}_{F}=\left\{B_{2},B_{3},B_{5},B_{6}\right\}$.

**Definition** **6.** *In light of Definition 5, we define a new state set based on the ideal inferiority class as follows:*

$$\begin{array}{c} G1=\left\{O_{i}|\frac{|{\left[O_{i}\right]}_{F}|}{\left|n\right|}\geq k_{2}\right\},\;G2=\left\{O_{i}|\frac{|{\left[O_{i}\right]}_{F}|}{\left|n\right|}<k_{2}\right\},k_{2}\in \left[0.5,1\right], \end{array}$$

where $|{\left[O_{i}\right]}_{F}|$ is the cardinality of the ideal inferiority class of $O_{i}$, and $k_{2}$ is the preference parameter of the ideal inferiority class decided by decision-makers; we set the value range of $k_{2}$ to be between 0.5 and 1. G1 represents “excellent” objects; then, G2 represents “non-excellent” objects. If the cardinality of the inferiority class of $O_{i}$ divided by the total number of objects equals the preference parameter $k_{2}$, then we divide object $O_{i}$ into the set of “excellent” objects. The formula for calculating conditional probability under the ideal inferiority class is $P\left(⊙|{\left[O_{i}\right]}_{F}\right)=\frac{|⊙⋂{\left[O_{i}\right]}_{F}|}{|{\left[O_{i}\right]}_{F}|},\left(⊙=\left\{G1,G2\right\}\right)$.

**Example** **5.** *(Continued from Example 4) Let $k_{2}=0.5$. In line with Definition 6, we can obtain the state set of the ideal inferiority class as follows:*

$$\begin{array}{c} G1=\left\{B_{1},B_{4},B_{6}\right\},G2=\left\{B_{2},B_{3},B_{5}\right\}. \end{array}$$

When the ideal inferiority class and state set for each object are known, then, we calculate the conditional probability of each object in different states under the ideal inferiority class. In the *G*1 state, the conditional probabilities of each ideal inferiority class object are as follows:

$P\left(G1|{\left[B_{1}\right]}_{F}\right)=\frac{|G1⋂{\left[B_{1}\right]}_{F}|}{|{\left[B_{1}\right]}_{F}|}=\frac{|\left\{B_{1},B_{4},B_{6}\right\}⋂\left\{B_{1},B_{2},B_{3},B_{5},B_{6}\right\}|}{\left|\{B_{1},B_{2},B_{3},B_{5},B_{6}\}\right|}=\frac{2}{5}$.

$P\left(G1|{\left[B_{2}\right]}_{F}\right)=\frac{|G1⋂{\left[B_{2}\right]}_{F}|}{|{\left[B_{2}\right]}_{F}|}=\frac{|\left\{B_{1},B_{4},B_{6}\right\}⋂\left\{B_{2}\right\}|}{\left|\{B_{2}\}\right|}=0$.

$P\left(G1|{\left[B_{3}\right]}_{F}\right)=\frac{|G1⋂{\left[B_{3}\right]}_{F}|}{|{\left[B_{3}\right]}_{F}|}=\frac{|\left\{B_{1},B_{4},B_{6}\right\}⋂\left\{B_{2},B_{3}\right\}|}{\left|\{B_{2},B_{3}\}\right|}=0$.

$P\left(G1|{\left[B_{4}\right]}_{F}\right)=\frac{|G1⋂{\left[B_{4}\right]}_{F}|}{|{\left[B_{4}\right]}_{F}|}=\frac{|\left\{B_{1},B_{4},B_{6}\right\}⋂\left\{B_{1},B_{2},B_{3},B_{4},B_{5},B_{6}\right\}|}{\left|\{B_{1},B_{2},B_{3},B_{4},B_{5},B_{6}\}\right|}=\frac{1}{2}$.

$P\left(G1|{\left[B_{5}\right]}_{F}\right)=\frac{|G1⋂{\left[B_{5}\right]}_{F}|}{|{\left[B_{5}\right]}_{F}|}=\frac{|\left\{B_{1},B_{4},B_{6}\right\}⋂\left\{B_{2},B_{5}\right\}|}{\left|\{B_{2},B_{5}\}\right|}=0$.

$P\left(G1|{\left[B_{6}\right]}_{F}\right)=\frac{|G1⋂{\left[B_{6}\right]}_{F}|}{|{\left[B_{6}\right]}_{F}|}=\frac{|\left\{B_{1},B_{4},B_{6}\right\}⋂\left\{B_{2},B_{3},B_{5},B_{6}\right\}|}{\left|\{B_{2},B_{3},B_{5},B_{6}\}\right|}=\frac{1}{4}$.

   In the *G*2 state, the ideal inferiority class conditional probability of each object can be obtained in the same way.

#### 3.2.2. The Three-Way Process Based on the Ideal Inferiority Class

For the ideal inferiority class, there is a similar DTRS process. When an object $O_{i}$ belongs to the state *G*1, the relative losses of selecting the action $L_{P}$, $L_{B}$ and $L_{N}$ are 0, $\theta_{{\left[O_{i}\right]}_{F}}^{{\left(BG1\right)}^{*}}$ and $\theta_{{\left[O_{i}\right]}_{F}}^{{\left(NG1\right)}^{*}}$ respectively. Likewise, when $O_{i}$ belongs to *G*2, the relative losses of selecting the action $L_{P}$, $L_{B}$ and $L_{N}$ are $\theta_{{\left[O_{i}\right]}_{F}}^{{\left(PG2\right)}^{*}}$, $\theta_{{\left[O_{i}\right]}_{F}}^{{\left(BG2\right)}^{*}}$ and 0, respectively. Similar to the ideal superiority class, there is also a reasonable hypothesis with practical significance for the ideal inferiority class:

$0\leq \theta_{{\left[O_{i}\right]}_{F}}^{{\left(BG1\right)}^{*}}<\theta_{{\left[O_{i}\right]}_{F}}^{{\left(NG1\right)}^{*}}$,

$0\leq \theta_{{\left[O_{i}\right]}_{F}}^{{\left(BG2\right)}^{*}}<\theta_{{\left[O_{i}\right]}_{F}}^{{\left(PG2\right)}^{*}}$.

   On the basis of Definitions 5 and 6, the expected losses of $O_{i}$ are divided into the positive territory, the boundary territory and the negative territory, which are computed as follows:

(24) $$Q\left(L_{P}|{\left[O_{i}\right]}_{F}\right)=\theta_{{\left[O_{i}\right]}_{F}}^{{\left(PG2\right)}^{*}}P\left(G2|{\left[O_{i}\right]}_{F}\right),$$

(25) $$Q\left(L_{B}|{\left[O_{i}\right]}_{F}\right)=\theta_{{\left[O_{i}\right]}_{F}}^{{\left(BG1\right)}^{*}}P\left(G1|{\left[O_{i}\right]}_{F}\right)+\theta_{{\left[O_{i}\right]}_{F}}^{{\left(BG2\right)}^{*}}P\left(G2|{\left[O_{i}\right]}_{F}\right),$$

(26) $$Q\left(L_{N}|{\left[O_{i}\right]}_{F}\right)=\theta_{{\left[O_{i}\right]}_{F}}^{{\left(NG1\right)}^{*}}P\left(G1|{\left[O_{i}\right]}_{F}\right).$$

In Table 11, we give the aggregate relative losses of $O_{i}$ based on *G*1 and *G*2.

In Table 11, $v_{max}=\max_{j\in \mu }v_{ij}$, $v_{min}=\min_{j\in \mu }v_{ij}$, and the risk avoidance coefficient τ complies with the requirement τ∈(0,0.5).

In light of the aggregate relative loss functions exhibited in Table 5, the thresholds $\alpha_{i}^{F},\beta_{i}^{F}\;\mathrm{and}\;\gamma_{i}^{F}$ of $O_{i}$ under the ideal inferiority relation *F* can be calculated as below:

(27) $$\alpha_{i}^{F}=\frac{\Sigma_{j}w_{j}\left(1-\tau \right)\left(v_{\max}-v_{ij}\right)}{\Sigma_{j}w_{j}\left(1-\tau \right)\left(v_{\max}-v_{ij}\right)+(\Sigma_{j}w_{j}\tau \left(v_{ij}-v_{\min}\right)},$$

(28) $$\beta_{i}^{F}=\frac{\Sigma_{j}w_{j}\tau \left(v_{\max}-v_{ij}\right)}{\Sigma_{j}w_{j}\tau \left(v_{\max}-v_{ij}\right)+\Sigma_{j}w_{j}\left(1-\tau \right)\left(v_{ij}-v_{\min}\right)},$$

(29) $$\gamma_{i}^{F}=\frac{\Sigma_{j}w_{j}\left(v_{\max}-v_{ij}\right)}{v_{\max}-v_{\min}}.$$

Finally, we give the TWD rules of the ideal inferiority class directly as follows:

$$\begin{array}{cc} \left(P7\right)\;\mathrm{If}\; & P\left(G1|{\left[O_{i}\right]}_{F}\right)\geq \alpha_{i}^{F},\mathrm{then}\;O_{i}\in POS\left(G1\right),\\ \left(B7\right)\;\mathrm{If}\; & \beta_{i}^{F}<P\left(G1|{\left[O_{i}\right]}_{F}\right)<\alpha_{i}^{F},\mathrm{then}\;O_{i}\in BND\left(G1\right),\\ \left(N7\right)\;\mathrm{If}\; & P\left(G1|{\left[O_{i}\right]}_{F}\right)\leq \beta_{i}^{F},\mathrm{then}\;O_{i}\in NEG\left(G1\right). \end{array}$$

**Example** **6.** *(Continued from Examples 4 and 5) In the tableware color selection problem, we set the risk-aversion coefficient of five attributes as τ={0.2,0.2,0.2,0.2,0.2}; next, we take object $B_{1}$ as an example and give the detailed Steps of the three-way process based on the ideal inferiority class.*

In the ideal inferiority class model, object $B_{1}$ is in two different states, and the relative loss functions of the three different behaviors have been obtained in Table 12, so now we solve the respective thresholds of object $B_{1}$.

$$\begin{array}{cccc} \; & \alpha_{1}^{F}=\frac{0.0762}{0.0762+0.0158}=0.8283,\; & \beta_{1}^{F}=\frac{0.0191}{0.0191+0.0631}=0.2323,\; & \gamma_{1}^{F}=\frac{0.0953}{0.1742}=0.5471. \end{array}$$

In Example 5, we calculated $P(G1|{\left[B_{1}\right]}_{F})=0.4000$; it can be seen that the conditional probability of object $B_{1}$ is less than $\alpha_{1}^{F}$ and greater than $\beta_{1}^{F}$; then, using the rules of (P7)–(N7), object $B_{1}$ is divided into the boundary territory. Similarly, the thresholds and classification results of other objects are in Table 13:

### 3.3. Description of the above Two Models

There are similarities and differences in the two TWD models constructed by using the TOPSIS method (the TWD ideal superiority model and the TWD ideal inferiority model). Differences: the definitions of the two models are definitely different and opposite; due to the different definitions of ideal classes, the definitions of state sets in the two models are also different. Similarities: the two models are defined using the core ideas of TOPSIS, and both of these models use relative loss functions.

All objects are classified into three regions according to the relationship between the thresholds α,β,γ and conditional probabilities $P(⋆|\left[O_{i}\right])$. Regarding the ordering of objects, objects in each domain are sorted according to their expected losses, and objects with lower expected losses are at the top of the list in these three regions. The expected loss represents the cost of an action, so when the object performs an action, the lower the loss, the better, which means that the action is a possible desirable decision. Finally, based on POS(⋆)≻BNG(⋆)≻NEG(⋆), where ⋆={T1,T2,G1,G2} and ≻ is the superiority relationship, we can obtain the ranking of all objects.

## 4. An Application of the Proposed TWD-MADM Approach

We apply the new TWD models established in Section 3 to a concrete application example, namely the selection of building appearances. The dataset is selected from the UCI database. Meanwhile, we provide decision algorithms based on the models, as well as the time complexity of those algorithms.

### 4.1. Introduction of the Problem

In our daily lives, we might see a wide range of building appearances. Buildings come in a variety of shapes and sizes, some for functional purposes, some for aesthetic reasons and some to represent regional culture. Real estate decision-makers must select the best out of numerous building look possibilities to develop in a certain location by taking into account a wide range of features, which is an MADM issue.

Buildings differ with respect to glazing area, glazing area distribution and orientation, among other parameters. We simulate various settings as functions of the aforementioned characteristics to obtain 768 building shapes. The dataset is comprised of 768 samples and 8 features. Let I=(O,S) be an information system for building shape selection, where $O_{i}\in O\left(i\in \eta \right)$ stands for the set of building shapes and $S_{j}\in S\left(j\in \mu \right)$ represents the set of building attributes. In this example, we have a total of eight different building attributes. $S_{1}$ stands for “ Relative Compactness”. $S_{2}$ stands for “Surface Area”. $S_{3}$ stands for “Wall Area”. $S_{4}$ stands for “Roof Area”. $S_{5}$ stands for “Overall Height”. $S_{6}$ stands for “Orientation”. $S_{7}$ stands for “ Glazing Area”. $S_{8}$ stands for “ Glazing Area Distribution”. The original data for building shape selection are shown in Table 14.

### 4.2. The Decision-Making Algorithms

In order to illustrate the correctness of the application of our proposed models in MADM, Algorithms 1 and 2 show the detailed decision-making processes of the building appearance selection problem.

The decision process of Algorithm 1 based on the ideal superiority class is as follows:
**Algorithm 1:** Decision-making algorithm based on the ideal superiority class**Input:***I* information system, *W* weight, τ risk avoidance coefficient.**Output:** The optimal building shape and the order of all building shapes.**Step 1:** Choose different normalization formulas to normalize all of the evaluation values based on the nature of the attributes.**Step 2:** Calculate the BTID and the WTID of each object by using Formula (10).**Step 3:** Find the ideal superiority class of each object by Formula (12).**Step 4:** For each object, determine whether it is in the T1 or T2 state by Definition 3.**Step 5:** Compute the conditional probability of each object by Remark 2.**Step 6:** Calculate the loss function θ by using the evaluation value $v_{ij}$, weight *W* and risk avoidance coefficient τ in the information matrix, and then use Formulas (19)–(21) to obtain the three thresholds $\alpha^{E}$, $\beta^{E}$ and $\gamma^{E}$.**Step 7:** Divide all objects into three regions according to (P6)–(N6), the relationship between thresholds and conditional probabilities.**Step 8:** Use Formulas (16)–(18) to obtain the expected loss of each object.**Step 9:** Sort objects in each region according to the expected losses, and then derive the optimal building shape and the order of all shapes based on POS(⋆)≻BNG(⋆)≻NEG(⋆).

**Remark** **4.** *Analysis of the time complexity of each step of the above algorithm: The first step is to normalize each evaluation value with a time complexity O(n). Step 2 computes the BTID and WTID for each object, with a time complexity of O(n). Step 3 is to determine the ideal superiority class of each object, with a time complexity of $O(n^{2})$. The fourth step is to determine whether each object is in the T1 or T2 state, with a time complexity of O(n). Step 5 calculates the conditional probability of each object in the ideal superiority class using the conditional probability formula, with a time complexity O(n). Step 6 computes the thresholds for each object with a time complexity O(n). Step 7 is to divide all objects into three areas according to the TWD rules, with a time complexity O(n). Step 8 computes the expected loss of each object with a time complexity O(n). Step 9 sorts all objects according to POS(⋆)≻BNG(⋆)≻NEG(⋆), which has a time complexity O(nlogn). Therefore, the time complexity of the entire algorithm is $O(n^{2})$.*

The decision process of Algorithm 2 based on the ideal inferiority class is as follows:
**Algorithm 2:** Decision-making algorithm based on the ideal inferiority class**Input:***I* information system, *W* weight, τ risk avoidance coefficient.**Output:** The optimal building shape and the order of all building shapes.**Step 1:** Choose different normalization formulas to normalize all of the evaluation values based on the nature of the attributes.**Step 2:** Compute the BTID and the WTID of each object by using Formula (10).**Step 3:** Find the ideal inferiority class of each object by using Formula (23).**Step 4:** For each object, determine whether it is in the G1 or G2 state by Definition 6.**Step 5:** Compute the conditional probability of each object by the conditional probability formula of the ideal inferiority class $P(⊙|{\left[O_{i}\right]}_{F}),i\in \eta$.**Step 6:** Calculate the loss function θ, and then use Formulas (27)–(29) to compute the three thresholds $\alpha^{F}$, $\beta^{F}$ and $\gamma^{F}$.**Step 7:** Divide all objects into three regions according to (P7)–(N7).**Step 8:** Obtain the expected loss of each object via Formulas (24)–(26).**Step 9:** Sort objects in each region according to the expected losses, and then derive the optimal building shape and the order of all shapes based on POS(⋆)≻BNG(⋆)≻NEG(⋆).

**Remark** **5.** *Similar to the analysis of Algorithm 1, we obtain that the time complexity of Algorithm 2 is also $O(n^{2})$.*

### 4.3. An Application Example

We use our proposed algorithms to address the problem of architectural shape selection in order to demonstrate the practicality of our proposed models. Consider a scenario where a developer purchases a block of land with the intention of developing a retro-style residential district comprised entirely of single-family apartments. Since the overall height of the buildings must be consistent for coordination and aesthetics, we need not consider $S_{5}$ (Overall Height). Because of the retro style, $S_{7}$ (Glazing Area) and $S_{8}$ (Glazing Area Distribution) are not taken into account either. Meanwhile, we randomly select eight objects from Example 4 for the sake of simplicity. Consequently, we use $O=\{O_{1},O_{2},O_{3},O_{4},O_{5},O_{6},O_{7},O_{8}\}$ to represent the set of eight building shapes, and $S=\{S_{1},S_{2},S_{3},S_{4},S_{5}\}$ to represent the set of five building attributes (i.e., Relative Compactness, Surface Area, Wall Area, Roof Area and Orientation), while the weights of the five attributes above are 0.2, 0.1, 0.3, 0.2 and 0.2, respectively. Among all attributes, $S_{3}$ is an expense attribute, and the rest are profit attributes. The risk-aversion coefficient for each attribute is 0.35, and the overall dataset is shown in Table 15.

First, we must normalize the values in Table 15 using the following two formulas:

(30) $$v_{ij}=\frac{u_{ij}}{\max_{i\in \eta }u_{ij}},S_{j}\;\mathrm{is}\;\mathrm{the}\;\mathrm{profit}\;\mathrm{attribute}.$$

(31) $$v_{ij}=\frac{\min_{i\in \eta }u_{ij}}{u_{ij}},S_{j}\;\mathrm{is}\;\mathrm{the}\;\mathrm{expense}\;\mathrm{attribute}.$$

The normalized results are shown in Table 16.

**(1) The decision-making process using the TWD model based on the ideal superiority class.**

By means of the TOPSIS method and Definition 1, we can obtain the following list of ideal superiority classes for all objects:

${\left[O_{1}\right]}_{E}=\left\{O_{1},O_{2},O_{4},O_{5},O_{6},O_{7}\right\}$

${\left[O_{2}\right]}_{E}=\left\{O_{2},O_{7}\right\}$

${\left[O_{3}\right]}_{E}=\left\{O_{1},O_{2},O_{3},O_{4},O_{5},O_{6},O_{7}\right\}$

${\left[O_{4}\right]}_{E}=\left\{O_{1},O_{2},O_{4},O_{5},O_{6},O_{7}\right\}$

${\left[O_{5}\right]}_{E}=\left\{O_{5},O_{7}\right\}$

${\left[O_{6}\right]}_{E}=\left\{O_{6},O_{7}\right\}$

${\left[O_{7}\right]}_{E}=\left\{O_{7}\right\}$

${\left[O_{8}\right]}_{E}=\left\{O_{1},O_{2},O_{4},O_{5},O_{6},O_{7},O_{8}\right\}$.

After obtaining the ideal superiority class of each object, we know the number of objects each object is superior to; the next step is to compute the state set by utilizing our newly established state set method. In the process of solving the state set, we divide all objects into two states, T1 or T2. The specific steps are in Step 4 of Algorithm 1, and the results are as follows (the preference parameter $k_{1}=0.2$ in this project):

$T1=\{O_{7}\}$

$T2=\{O_{1},O_{2},O_{3},O_{4},O_{5},O_{6},O_{8}\}$.

Knowing the set of ideal superiority classes and state set, we can calculate the conditional probabilities for all building shapes; the specific calculation results are shown in Table 17.

According to Table 7, we work out the relative loss function of each building shape taking three actions in two different states. To further calculate the threshold of each building shape, by using the rules of (P6)–(N6) and comparing the conditional probability of each building shape with its three thresholds $\alpha^{E},\beta^{E}$ and $\gamma^{E}$, each building shape can be divided into a specific domain. The specific thresholds and partition results of the eight building shapes are shown in Table 18.

In the end, the objects in each domain are ordered based on their expected losses: the lower the expected loss, the higher the ranking. The ranking between domains can be obtained from the positive territory being superior to the boundary territory, and the boundary territory being superior to the negative territory. The specific ranking results are shown in Table 19.

From Table 19, we know that the building shape classified into the positive domain of TWD is $O_{7}$, which is the optimal building shape in this project. For $O_{2}$, $O_{5}$ and $O_{6}$ divided in the boundary domain of TWD, further consideration and evaluation are needed before making a decision. At the same time, it is also provided that $O_{2}$ is given priority over $O_{5}$, and $O_{5}$ is given priority over $O_{6}$. Whereas $O_{3}$, $O_{8}$, $O_{1}$ and $O_{4}$ are divided in the negative domain of TWD and can be discarded. Hence, under the ideal superiority class model, developers can give priority to building shape $O_{7}$ in the project.

**(2) The decision-making process using the TWD model based on the ideal inferiority class**

Through the TOPSIS method and Definition 4, we can obtain the following list of ideal inferiority classes for all objects:

${\left[O_{1}\right]}_{F}=\left\{O_{1},O_{3},O_{4},O_{8}\right\}$

${\left[O_{2}\right]}_{F}=\left\{O_{1},O_{2},O_{3},O_{4},O_{8}\right\}$

${\left[O_{3}\right]}_{F}=\left\{O_{3}\right\}$

${\left[O_{4}\right]}_{F}=\left\{O_{1},O_{3},O_{4},O_{8}\right\}$

${\left[O_{5}\right]}_{F}=\left\{O_{1},O_{3},O_{4},O_{5},O_{8}\right\}$

${\left[O_{6}\right]}_{F}=\left\{O_{1},O_{3},O_{4},O_{6},O_{8}\right\}$

${\left[O_{7}\right]}_{F}=\left\{O_{1},O_{2},O_{3},O_{4},O_{5},O_{6},O_{7},O_{8}\right\}$

${\left[O_{8}\right]}_{F}=\left\{O_{8}\right\}$.

Having obtained the ideal inferiority class of each object, we need to compute the state under the ideal inferiority class by using Step 4 in Algorithm 2, and then divide these eight objects into two states, i.e., *G*1 or *G*2 (the preference parameter $k_{2}=0.5$ in this project).

$G1=\{O_{1},O_{2},O_{4},O_{5},O_{6},O_{7}\}$

$G2=\{O_{3},O_{8}\}$.

Once the set of ideal inferiority classes and state set are known, we can separately compute the conditional probabilities for these eight building shapes; the results are shown in Table 20.

Likewise, according to Table 11, we can work out the relative loss function of taking three behaviors respectively when the object is in *G*1 or *G*2 state, and the threshold value of each object is further obtained by using the rules of (27)–(29). Then, we can divide each object into a clear domain via using the division rule of TWD, which is (P7)–(N7). Table 21 shows the thresholds and decision rules for the eight building shapes under the TWD ideal inferiority model.

Finally, we categorize the eight objects into three domains. In each domain, we rank the objects by their own expected loss: the smaller the expected loss, the higher the object ordering. The ordering between domains is given by POS(⋆)≻BNG(⋆)≻NEG(⋆), and the final ranking results are shown in Table 22.

From Table 22, we know that $O_{7}$, $O_{2}$ and $O_{6}$ are in the positive domain, and $O_{7}$ is superior to $O_{2}$ and $O_{2}$ is superior to $O_{6}$, thus the optimal building shape under the TWD ideal inferiority class model is also $O_{7}$. In the boundary domain, there are three objects, $O_{1}$, $O_{4}$ and $O_{5}$. In the negative domain, there are two objects, $O_{3}$ and $O_{8}$.

In comparison to the TWD ideal superiority model, we can analyze from three aspects: the optimal object, the partial ordering and the classification of objects. First of all, the optimal building shape for both models is $O_{7}$, which shows the two models are consistent in selecting the optimal object. Afterwards, the partial ordering of the two models is consistent to a certain extent, such as the ordering of the first three objects is the same: $O_{7}≻O_{2}≻O_{6}$; further, $O_{1}≻O_{4}$ and $O_{3}≻O_{8}$. In the end, for the classification of objects, some objects are divided into the same domain in the two models. For instance, $O_{7}$ is divided into the positive domain, $O_{5}$ is divided into the boundary domain, and $O_{3}$ and $O_{8}$ are divided into the negative domain. Analysis and comparison of the above three aspects shows that the two new models proposed in this paper are consistent to a certain extent.

### 4.4. Comparison Analysis and Spearman’s Rank Correlation Analysis

In the following, we compare and analyze the ranking results of the two proposed models with five other MADM methods.

#### 4.4.1. Comparison Analysis of Different MADM Approaches

In order to verify the effectiveness and reasonableness of the models we proposed, we take the example in Section 4.3 to compare and analyze the ranking results of our models with five other MADM methods: TOPSIS [2], PROMETHEE [22], Ye’s method [11], Zhang’s method [23] and Jia’s method [3]. The specific ranking results obtained from the above methods are shown in Table 23 below:

In Table 23, IS represents the TWD ideal superiority model, and IF represents the TWD ideal inferiority model. For these seven MADM methods, we implement a comprehensive analysis and discussion from three perspectives, namely overall ranking analysis, partial ranking comparison and selection of the optimal object.

(a) From the perspective of overall ranking, we can conclude that the seven MADM methods all give the ranking results of the eight objects, but compared with the traditional TOPSIS and PROMETHEE methods, the two proposed methods not only give the ranking of the objects, but also the classification of the objects. Further, we can see that Jia’s method and the PROMETHEE method yield identical ranking results, $O_{7}≻O_{2}≻O_{5}≻O_{6}≻O_{1}≻O_{4}≻O_{8}≻O_{3}$, and other MADM methods all have sorting differences. Our proposed IS method and Zhang’s method have the same ordering of the first four objects.

(b) From the perspective of the partial ranking comparison, the ordering of $O_{1}$ and $O_{4}$ in all MADM methods is the same as $O_{1}≻O_{4}$. In seven of the MADM methods (in all but Ye’s method), the ranking position of $O_{2}$ is second to the best option $O_{7}$. In Ye’s method, the second sorting position is $O_{5}$. Hence, $O_{2}$ and $O_{5}$ are either divided into the positive territory or the boundary territory. Furthermore, $O_{3}$, $O_{4}$ and $O_{8}$ are the worst selections, which are sorted at the bottom in all methods. Moreover, $O_{3}$ is ranked in the last position in TOPSIS, PROMETHEE, Zhang’s and Jia’s, $O_{8}$ is ranked in the last position in IF and Ye’s, and $O_{4}$ is ranked in the last position in IS. This means the approximate range of sorting positions for all objects is the same.

(c) From the perspective of the selection of the optimal object, it is not difficult to find that the best choice of our two proposed methods and other MADM methods are $O_{7}$, and in our proposed methods, $O_{7}$ is classified into the positive domain, which shows that our proposed method and other methods are consistent in the selection of the best object, and also shows the feasibility of our proposed method.

In general, Table 23 shows that our proposed models are consistent with the five other MADM methods, and our method not only has the sorting results, but also the classification of each object. From the overall analysis and discussion, our proposed method has certain feasibility and rationality.

#### 4.4.2. Spearman’s Rank Correlation Analysis

In order to analyze and compare the correlations and differences between the selected MADM methods, we use the Spearman’s correlation coefficient (SRCC) as an indicator. The SRCC is calculated as follows:

(32) $$SRCC=1-\frac{6\Sigma_{i=1}^{n}q_{i}^{2}}{n^{3}-n},$$

where *n* is the total number of objects, and $q_{i}=x_{i}-y_{i}$, in which $x_{i}$ is the ranking position of $O_{i}$ in one MADM method and $y_{i}$ is the ranking position of $O_{i}$ in another MADM method. If the SRCC of the two ranking results is greater, then we may say that they are highly relevant and consistent between the two decision-making methods. That is, for any two decision-making methods after processing the same data, the larger the correlation coefficient value of the SRCC, the better the correlation and consistency between the two methods. On the basis of Table 23, we can calculate the SRCC between any two different methods, as shown in Table 24.

**Figure 1:**
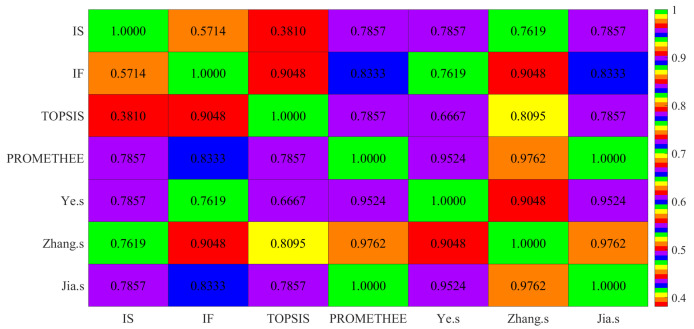
The heatmap of SRCC in seven multi-criteria decision-making methods.

To visualize the SRCC and enhance the readability of the data, this paper uses a heatmap, which is a matrix that shows the data in terms of color changes that represent the magnitude of the correlation coefficient, thus showing the correlation between different indicators and different samples. The heatmap of Table 24 described above is shown in Figure 1.

From Table 24, for the IS method we proposed, the SRCC values of IF and TOPSIS method is low, only 0.5714 and 0.3810, respectively; however, the SRCC values between it and PROMETHEE, Ye’s method, Zhang’s method and Jia’s method are all greater than 0.648, indicating that the IS method has certain rationality and feasibility. For our proposed IF method, the SRCC values of it and the other five MADM methods are all greater than 0.648, and the lowest value is 0.7619 with the Ye’s method; the SRCC value with the TOPSIS and Zhang’s methods is as high as 0.9048, which shows that the IF method has strong consistency with all other MADM methods. Further, the two methods we proposed also have certain differences in the SRCC value. Compared with the IS method, the IF method has higher consistency with other MADM methods. On the whole, the proposed method has high consistency and similarity with other MADM methods.

#### 4.4.3. Other Example Analysis

In order to verify the high reliability and practicability of our proposed model, we additionally cite two sets of data from [3,16]; then, the results of the proposed method are compared and analyzed with those of other MADM methods.

**Example** **7.** *A classic corporate investment problem. There are eight investment objects $O=\{O_{1},O_{2},O_{3},O_{4},O_{5},O_{6},O_{7},O_{8}\}$ and five attributes $S=\{S_{1},S_{2},S_{3},S_{4},S_{5}\}$, i.e., expected benefits, environmental influence, market saturation, social benefits and energy conservation, respectively. Among them $S_{2}$ and $S_{3}$ are cost attributes, and $S_{1},S_{4}$ and $S_{5}$ are benefit attributes. The weight vectors of these five attributes are w={0.3,0.1,0.3,0.2,0.1}, and the risk avoidance coefficient vectors are τ={0.1,0.1,0.1,0.1,0.1}. Consequently, the specific relevant data of eight investment objects over five attributes are presented in Table 25.*

**Figure 2:**
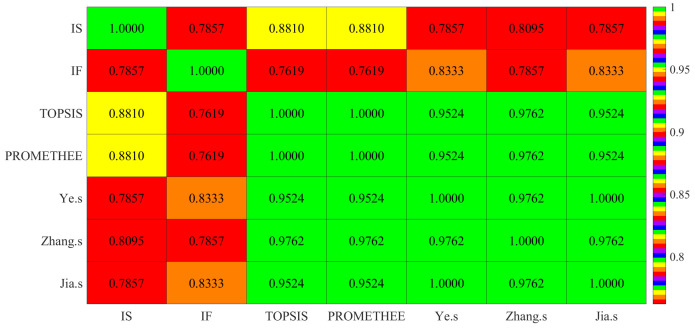
The heatmap of SRCC in seven multi-criteria decision-making methods.

Using the data in Table 25, we conduct a comprehensive analysis and discussion of the results obtained by our two proposed methods and those obtained by other MADM methods. The ranking results for these eight objects in different MADM methods are presented in Table 26, and the SRCC values for each method in relation to the ranking results are shown in Table 27. In order to visualize SRCC, the heatmap of SRCC values in Table 27 is shown in Figure 2.

**Remark** **6.** *From the ranking results in Table 26, the optimal objects selected by our proposed methods and other MADM methods are uniform: $O_{7}$. The worst objects are $O_{3}$ and $O_{8}$. In IS, TOPSIS, PROMETHEE and Zhang’s, the worst object is $O_{3}$, while in IF, Ye’s and Jia’s, the worst object is $O_{8}$. Moreover, Objects $O_{2}$ and $O_{4}$ cannot be prioritized and classified in Ye’s method, but in our proposed IS method, we conclude that $O_{2}$ is better than $O_{4}$, and they are both classified into the boundary domain. Likewise, in our proposed IF method, we can conclude that $O_{4}$ is better than $O_{2}$ and is also classified into the boundary domain. It shows that our method has more sorting and classification advantages over Ye’s. From Table 27, we can find that the SRCC values of the IS and IF methods and other existing MADM methods are all greater than 0.648, which shows that our method is consistent and feasible with existing MADM methods.*

**Example** **8.** *An energy selection program: there are six energy projects $O=\{O_{1},O_{2},O_{3},O_{4},O_{5},O_{6}\}$ and four attributes $S=\{S_{1},S_{2},S_{3},S_{4}\}$. Among them $S_{2}$ is a cost attribute, and $S_{1},S_{3}$ and $S_{4}$ are benefit attributes. The weight vectors of these four attributes are w={0.2,0.2,0.3,0.3}. The specific data of six energy projects over four attributes are presented in Table 28.*

In this energy project example, we choose to compare our two proposed methods with three other MADM methods: two classic methods, TOPSIS and PROMETHEE, and the state-of-the-art Zhang’s method. The ranking results of the five methods are shown in Table 29 following.

**Figure 3:**
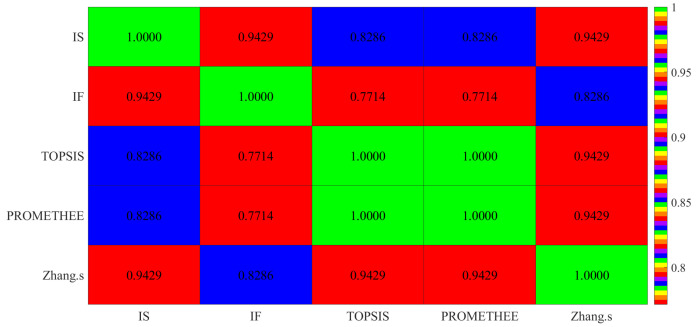
The heatmap of SRCC in five multi-criteria decision-making methods.

From the results in Table 29, the optimal project determined by our proposed methods and the three existing methods is identical: $O_{6}$. Moreover, from the perspective of overall ranking results, the ranking positions of objects in our method are generally similar to other methods, which implies that the proposed methods are credible and reasonable. To more clearly illustrate the connection and consistency between our proposed method and the TOPSIS, PROMETHEE and Zhang’s methods, we calculate the SRCC between any two MADM methods in Table 30 and Figure 3, and provide statistical significance ranking results for different methods.

## 5. Experiment Analysis

In this section, we conduct relevant experiment analyses on the adjustable parameters of our proposed models, including the preference parameter $k_{1}$ in the ideal superiority class model, the preference parameter $k_{2}$ in the ideal inferiority class model and the risk-aversion factor τ. Since we arbitrarily change the value of a parameter, the ranking and classification results of the decision will change, so it is very necessary to analyze and discuss the influence of the parameter value on the decision result. In the following, we continue to use the example in Section 4.3 to conduct experiments, the classification and ranking results in the two models are shown by varying the parameters $k_{1}$, $k_{2}$ in steps of 0.05 and the risk-aversion factor τ in steps of 0.1.

### 5.1. Analysis of the Preference Parameter $k_{1}$ and the Risk Aversion Factor τ in the Ideal Superiority Class Model

In each model, we have two variable parameters that are determined by the decision-maker according to the experimental situation. If the two parameters change at the same time, there will be countless possibilities. Therefore, we use the method of controlling variables to implement the experiment.

$\left(1\right)$ In the first case, we set the risk-aversion coefficient of each attribute to a fixed value and adjust the value of $k_{1}$ to obtain the following classification and sorting results for the eight objects in the TWD ideal superiority model. According to Definition 3, the value of $k_{1}$ ranges from 0 to 0.5 and changes with a step size of 0.05. In order to present our results more clearly and intuitively, we will show the sorting results of different values of $k_{1}$ in the form of a graph as follows.

**Figure 4:**
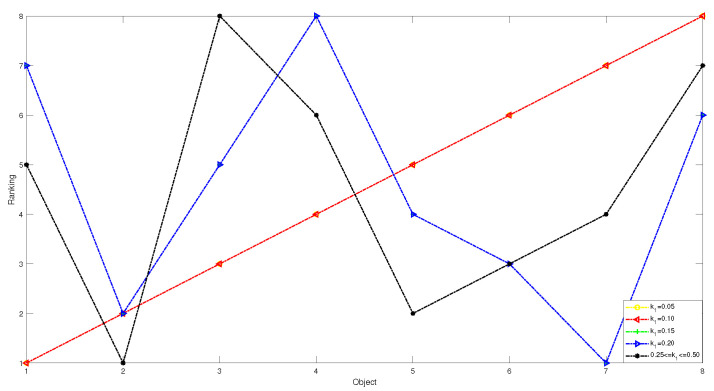
The sorting results of eight objects with different preference parameters $k_{1}$.

**Figure 5:**
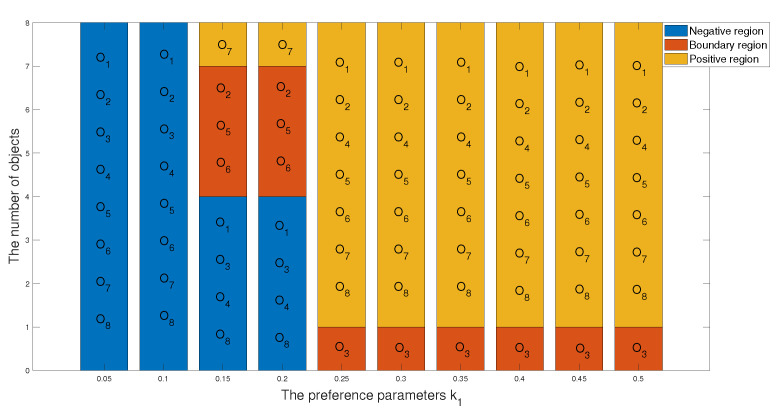
The classification results of eight objects with different preference parameters $k_{1}$.

**Remark** **7.** *From Table 31 and Figure 4 and Figure 5, we find that the results obtained for different values of $k_{1}$ will be different when $k_{1}$ is less than or equal to 0.1. There are no objects that meet the conditions. We can obtain that the objects in the $T_{1}$ state set are empty sets, so in the $T_{1}$ state set, the conditional probabilities of the eight objects are all 0, and it can be seen that they are all divided into the negative domain, and the ranking result is $O_{1}≻O_{2}≻O_{3}≻O_{4}≻O_{5}≻O_{6}≻O_{7}≻O_{8}$. In this project, due to the small number of objects, when the value of $k_{1}$ is less than or equal to 0.1, it is obviously unreasonable because it is impossible to reasonably distinguish and classify objects. When $k_{1}=0.15$ or $k_{1}=0.2$, the results are the same, and the broken line trajectories of the two values also coincide. This shows that the ideal number of superiority classes of each object is less than 2; that is, it satisfies the condition of $T_{1}$ state set: there is object $O_{7}$, and the conditional probability of each object is calculated to not be 0. The final ranking and classification results are highly consistent with other MADM methods. Specifically, the ranking result is $O_{7}≻O_{2}≻O_{5}≻O_{6}≻O_{3}≻O_{8}≻O_{1}≻O_{4}$, with $O_{7}$ in the positive domain, objects $O_{2}$, $O_{5}$ and $O_{6}$ in the boundary domain, and objects $O_{1},O_{3},O_{4}$ and $O_{8}$ in the negative domain. Furthermore, when $k_{1}$ is in [0.25, 0.5], we find that the classification and ranking results of the objects are identical, which means that the results tend to stabilize when $k_{1}$ reaches a certain threshold, and the ranking result after stabilization is $O_{2}≻O_{5}≻O_{6}≻O_{7}≻O_{1}≻O_{4}≻O_{8}≻O_{3}$, and $O_{1},O_{2},O_{4},O_{5},O_{6},O_{7},O_{8}$ is in the positive domain and $O_{3}$ is in the boundary domain.*

$\left(2\right)$ In the second case, we set the preference parameter $k_{1}$ with a fixed value and adjust the value of risk-aversion coefficient τ to obtain the following classification and sorting results for the eight objects in the TWD ideal superiority model.

**Figure 6:**
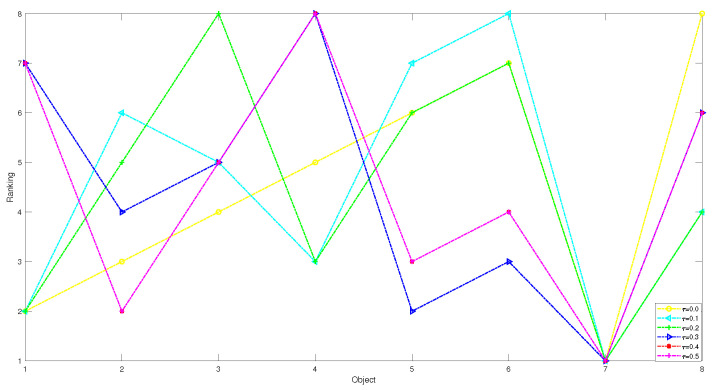
The sorting results of eight objects with different risk-aversion coefficients τ.

**Figure 7:**
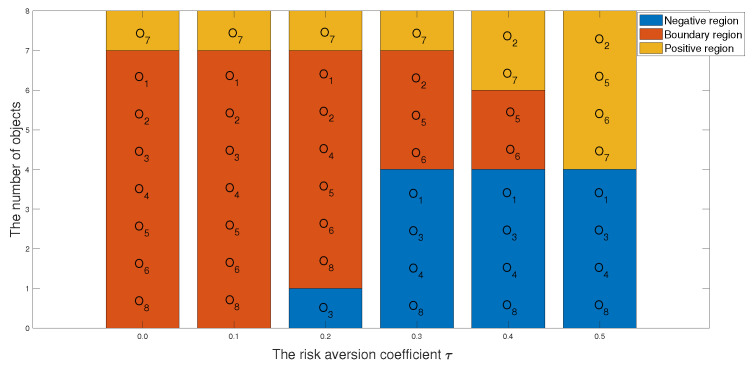
The classification results of eight objects with different risk-aversion coefficients τ.

**Remark** **8.** *From Table 32 and Figure 6 and Figure 7, we find that no matter what the value of τ is, $O_{7}$ is the optimal object and is in the positive domain. When τ = 0 and τ = 0.1, from the classification of Figure 7, the classification of the objects is the same, i.e., $O_{7}$ is in the positive domain, and objects $O_{1},O_{2},O_{3},O_{4},O_{5},O_{6},O_{8}$ are in the boundary domain. However, from the ranking of Figure 6, the ordering is different: for τ = 0, the ordering is $O_{7}≻O_{1}≻O_{2}≻O_{3}≻O_{4}≻O_{5}≻O_{6}≻O_{8}$, while for τ = 0.1, the ranking is $O_{7}≻O_{1}≻O_{4}≻O_{8}≻O_{3}≻O_{2}≻O_{5}≻O_{6}$. In contrast to the above, when τ = 0.4 and τ = 0.5, objects are ranked identically but classified differently: for τ = 0.4, $0_{5},O_{6}$ are classified into the boundary domain, but whenτ = 0.5, $0_{5},O_{6}$ is divided into the positive domain.*

### 5.2. Analysis of the Preference Parameter $k_{2}$ and the Risk Aversion Factor τ in the Ideal Inferiority Class Model

$\left(1\right)$ In the first case, we set the risk-aversion coefficient of each attribute to a fixed value and adjust the value of $k_{2}$ to obtain the following classification and sorting results for the eight objects in the TWD ideal inferiority model. According to Definition 6, the value of $k_{2}$ ranges from 0.5 to 1 and changes with a step size of 0.05. In order to present our results more clearly and intuitively, we will show the sorting results of different values of $k_{2}$ in the form of a graph as follows.

**Figure 8:**
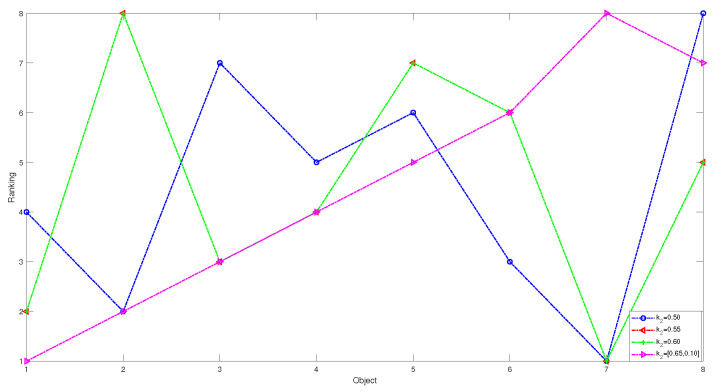
The sorting results of eight objects with different preference parameters $k_{2}$.

**Figure 9:**
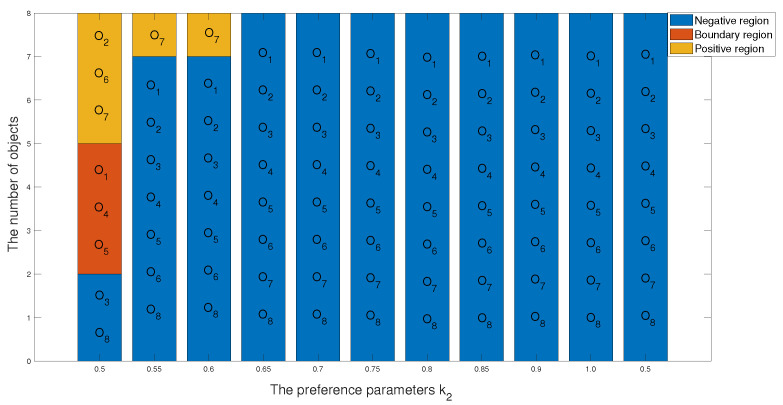
The classification results of eight objects with different preference parameters $k_{2}$.

**Remark** **9.** *According to Table 33 and Figure 8 and Figure 9, we derive the following information: when $k_{2}=0.50$, the sorting result is $O_{7}≻O_{2}≻O_{6}≻O_{1}≻O_{4}≻O_{5}≻O_{3}≻O_{8}$, in which the positive domain has objects $O_{2},O_{6}$ and $O_{7}$, the boundary domain has objects $O_{1},O_{4}$ and $O_{5}$, and the negative domain has objects $O_{3}$ and $O_{8}$. The value of $k_{2}$ is 0.5, indicating that the number of ideal inferiority classes of each object needs to be greater than or equal to half of the total number of objects before being classified into G1. From the perspective of this project, the ideal inferiority class with objects $O_{1},O_{2},O_{4},O_{5},O_{6},O_{7}$ satisfies the condition and is divided into the G1 state set. In the ideal inferiority class model, this value $k_{2}=0.50$ is reasonable and feasible, neither too harsh nor too loose. When $k_{2}$ is between 0.55 and 0.60, the ranking results are the same: $O_{7}≻O_{1}≻O_{3}≻O_{4}≻O_{8}≻O_{6}≻O_{5}≻O_{2}$, with only $O_{7}$ in the positive domain and the remaining objects in the negative domain. When $k_{2}$ is greater than or equal to 0.65 in the TWD ideal inferiority model, all objects are divided into the negative domain, and the sorting result is $O_{1}≻O_{2}≻O_{3}≻O_{4}≻O_{5}≻O_{6}≻O_{8}≻O_{7}$. In this example, when $k_{2}$ is 0.65, if the object is divided into G1, the ideal number of inferiority classes of the object must satisfy six or more, but from these eight objects, only $O_{7}$ satisfies the condition. Therefore, the ranking and classification results are consistent.*

$\left(2\right)$ In the second case, we set the preference parameter $k_{2}$ with a fixed value and adjust the value of risk-aversion coefficient τ to obtain the following classification and sorting results for the eight objects in the TWD ideal inferiority model.

**Figure 10:**
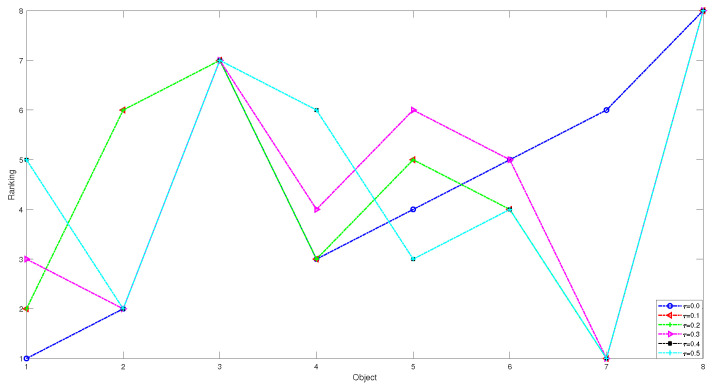
The sorting results of eight objects with different risk-aversion coefficients τ.

**Figure 11:**
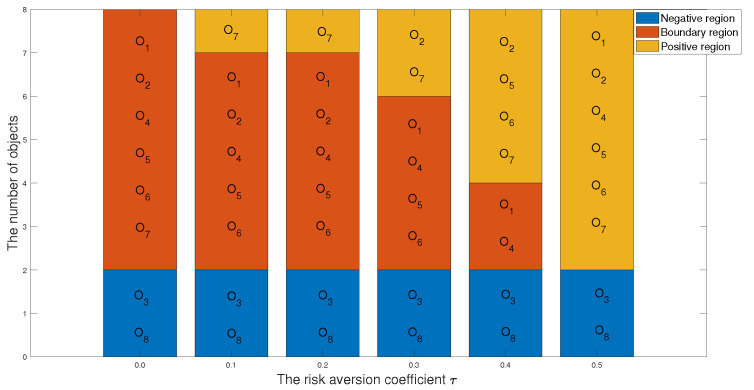
The classification results of eight objects with different risk-aversion coefficients τ.

**Remark** **10.** *According to Table 34 and Figure 10 and Figure 11, we analyze from the perspective of the optimal object, discovering that except for τ=0, the rest of the optimal objects are $O_{7}$. Furthermore, regardless of the value of τ, $O_{3}$ and $O_{8}$ are both classified into the negative domain, which indicate that $O_{3}$ and $O_{8}$ are not considered objects. When τ=0, that is, each attribute has no risk-aversion value, the final classification result is that there are no objects in the positive domain: $O_{1},O_{2},O_{4},O_{5},O_{6},O_{7}$ are in the boundary domain, and $O_{3}$ and $O_{8}$ are in the negative domain. The sort order is $O_{1}≻O_{2}≻O_{4}≻O_{5}≻O_{6}≻O_{7}≻O_{3}≻O_{8}$. When τ=0.1 and τ=0.2, the results of classification and ranking are identical. In Figure 10, it can be seen that the red line and the green line are coincident, and the sorting result is $O_{7}≻O_{1}≻O_{4}≻O_{6}≻O_{5}≻O_{2}≻O_{3}≻O_{8}$. In Figure 11, the division of the three areas of the histogram is the same: $O_{7}$ is in the positive domain, $O_{1},O_{2},O_{4},O_{5},O_{6}$ are in the boundary domain, and $O_{3}$ and $O_{8}$ are in the negative domain. When τ=0.4 and τ=0.5, although they are sorted the same, the classification is different. When τ=0.4, $O_{1}$ and $O_{4}$ are in the boundary domain; however, when τ=0.5, $O_{1}$ and $O_{4}$ are classified into the positive domain.*

## 6. Conclusions

In this study, we present two novel TOPSIS-based TWD models with opposing definitions: one of them is the TWD ideal superiority model, and the other is the TWD ideal inferiority model. When applied to practical fuzzy information systems, these two decision-making models demonstrate clear feasibility and rationality. In this paper, the datasets applicable to both models are fuzzy attribute environments. In addition, we propose a new method for objective computation of the state set that reduces the subjectivity of the decision process and makes the decision-making results more objective. Furthermore, we employ the relative loss functions of Jia and Liu to calculate the thresholds of each object, however, differently from Jia’s and Liu’s methods. Considering that it is subjective and undesirable to assign artificial, random values to each attribute of the risk-aversion factor, we set the risk avoidance coefficient of each attribute in the relative loss function to the same value. Moreover, due to the fact that this is a study of TWD in this paper, the risk-aversion coefficients range from 0 to 0.5, in spite of the values taken for each attribute being the same. Finally, according to the thresholds of each object and the TWD rules, all objects are divided into three different territories. The objects in the positive territory are acceptable, the objects in the boundary territory need further consideration, and the objects in the negative territory are rejected directly. Further, the objects in different domains are sorted according to the value of the loss function: the smaller the loss value, the more priority is given to sorting; finally, the sorting of all objects can be obtained, and it is known that the first object in the positive territory is the optimal object.

In the future, we will consider extending the applicability of the two models we proposed to other environments. Further, we can discuss and analyze the following three directions in depth: The first is to expand the TWD theories, which includes the expansion of relations, the expansion of related classification models and fusion with other classification methods. The second is the study of methodological aspects, such as decision-risk minimization and reduction methods, cost-sensitive rule acquisition and decision risk minimum rule acquisition. The third is the application of TWD in the fields of engineering, management and medicine.

## Figures and Tables

**Table 1:** The initial decision-making matrix.

	$S_{1}$	$S_{2}$	⋯	$S_{m}$
$O_{1}$	$u_{11}$	$u_{12}$	⋯	$u_{1m}$
$O_{2}$	$u_{21}$	$u_{22}$	⋯	$u_{2m}$
⋮	⋮	⋮	⋯	⋮
$O_{n}$	$u_{n1}$	$u_{n2}$	⋯	$u_{nm}$

**Table 2:** The normalized decision-making matrix.

	$S_{1}$	$S_{2}$	⋯	$S_{m}$
$O_{1}$	$v_{11}$	$v_{12}$	⋯	$v_{1m}$
$O_{2}$	$v_{21}$	$v_{22}$	⋯	$v_{2m}$
⋮	⋮	⋮	⋯	⋮
$O_{n}$	$v_{n1}$	$v_{n2}$	⋯	$v_{nm}$

**Table 3:** The relative loss functions of rule conversion.

	*R*	¬R
$L_{P}$	0	$\theta_{\prime }^{PN}$
$L_{B}$	$\theta_{\prime }^{BP}$	$\theta_{\prime }^{BN}$
$L_{N}$	$\theta_{\prime }^{NP}$	0

**Table 4:** The relative loss functions of rule conversion.

	$R_{j}$	$\neg R_{j}$
$L_{P}$	0	$v_{\max}^{j}-v_{ij}$
$L_{B}$	$\sigma (v_{ij}-v_{\min}^{j})$	$\sigma (v_{\max}^{j}-v_{ij})$
$L_{N}$	$v_{ij}-v_{\min}^{j}$	0

**Table 5:** The aggregate relative loss functions of $O_{i}$.

	*R*	¬R
$L_{P}$	0	$v_{\max}-\Sigma_{j}w_{j}v_{ij}$
$L_{B}$	$\Sigma_{j}\sigma_{j}w_{j}\left(v_{ij}-v_{\min}\right)$	$\Sigma_{j}\sigma_{j}w_{j}\left(v_{\max}-v_{ij}\right)$
$L_{N}$	$\Sigma_{j}w_{j}v_{ij}-v_{\min}$	0

**Table 6:** The multi-attribute information matrix of five funds.

	$r_{1}$	$r_{2}$	$r_{3}$	$r_{4}$
$s_{1}$	0.8	0.7	0.1	0.6
$s_{2}$	0.4	0.4	0.5	0.7
$s_{3}$	0.5	0.1	0.2	0.1
$s_{4}$	0.4	0.7	0.9	0.3
$s_{5}$	0.6	0.7	0.5	0.6

**Table 7:** The aggregate relative loss functions of $O_{i}\left(i\in \eta \right)$.

	T1	T2
$L_{P}$	0	$v_{\max}-\Sigma_{j}w_{j}v_{ij}$
$L_{B}$	$\Sigma_{j}\tau w_{j}\left(v_{ij}-v_{\min}\right)$	$\Sigma_{j}\tau w_{j}\left(v_{\max}-v_{ij}\right)$
$L_{N}$	$\Sigma_{j}w_{j}v_{ij}-v_{\min}$	0

**Table 8:** The aggregate relative loss functions of $s_{1}$ under the ideal superiority class.

	T1	T2
$L_{P}$	0	$v_{\max}-\Sigma_{j}w_{j}v_{1j}=0.5968$
$L_{B}$	$\Sigma_{j}\tau w_{j}\left(v_{1j}-v_{\min}\right)=0.1168$	$\Sigma_{j}\tau w_{j}\left(v_{\max}-v_{1j}\right)=0.2387$
$L_{N}$	$\Sigma_{j}w_{j}v_{1j}-v_{\min}=0.2921$	0

**Table 9:** The thresholds and division results of the other four objects.

	$s_{2}$	$s_{3}$	$s_{4}$	$s_{5}$
$\alpha_{i}^{E}$	0.6138	0.4410	0.6799	0.6456
$\beta_{i}^{E}$	0.4139	0.2596	0.4856	0.4473
$\gamma_{i}^{E}$	0.5144	0.3446	0.5861	0.5484
$P(T1|{\left[s_{i}\right]}_{E})$	0.2500	1.0000	0.5000	0.3333
Decision rules	NEG	POS	BND	NEG

**Table 10:** The multi-attribute information matrix of six tableware colors.

	$L_{1}$	$L_{2}$	$L_{3}$	$L_{4}$	$L_{5}$
$B_{1}$	0.86	588	294	147	2
$B_{2}$	0.82	602	118	121	3
$B_{3}$	0.76	701	356	145	5
$B_{4}$	0.90	637	343	165	2
$B_{5}$	0.86	588	294	118	3
$B_{6}$	0.82	634	348	163	3

**Table 11:** The aggregate relative loss functions of $O_{i}\left(i\in \eta \right)$.

	G1	G2
$L_{P}$	0	$v_{\max}-\Sigma_{j}w_{j}v_{ij}$
$L_{B}$	$\Sigma_{j}\tau w_{j}\left(v_{ij}-v_{\min}\right)$	$\Sigma_{j}\tau w_{j}\left(v_{\max}-v_{ij}\right)$
$L_{N}$	$\Sigma_{j}w_{j}v_{ij}-v_{\min}$	0

**Table 12:** The aggregate relative loss functions of $B_{1}$.

	G1	G2
$L_{P}$	0	$v_{\max}-\Sigma_{j}w_{j}v_{1j}=0.0953$
$L_{B}$	$\Sigma_{j}\tau w_{j}\left(v_{1j}-v_{\min}\right)=0.0158$	$\Sigma_{j}\tau w_{j}\left(v_{\max}-v_{1j}\right)=0.0191$
$L_{N}$	$\Sigma_{j}w_{j}v_{1j}-v_{\min}=0.0789$	0

**Table 13:** The thresholds and classification results of the five other objects.

	$B_{2}$	$B_{3}$	$B_{4}$	$B_{5}$	$B_{6}$
$\alpha_{i}^{F}$	0.7586	0.6929	0.5087	0.8607	0.6843
$\beta_{i}^{F}$	0.1642	0.1236	0.0608	0.2786	0.1193
$\gamma_{i}^{F}$	0.4400	0.3606	0.2056	0.6070	0.3514
$P(G1|{\left[B_{i}\right]}_{F})$	0.0000	0.0000	0.5000	0.0000	0.2500
Decision rules	NEG	NEG	BND	NEG	BND

**Table 14:** The original data for 385 building shapes.

	$S_{1}$	$S_{2}$	$S_{3}$	$S_{4}$	$S_{5}$	$S_{6}$	$S_{7}$	$S_{8}$
$O_{1}$	0.98	514.50	294.00	110.25	7.00	2	0.00	0
⋮	⋮	⋮	⋮	⋮	⋮	⋮	⋮	⋮
$O_{56}$	0.90	563.50	318.50	122.50	7.00	2	0.10	1
⋮	⋮	⋮	⋮	⋮	⋮	⋮	⋮	⋮
$O_{109}$	0.86	588.00	294.00	147.00	7.00	4	0.10	2
⋮	⋮	⋮	⋮	⋮	⋮	⋮	⋮	⋮
$O_{160}$	0.82	612.50	318.50	147.00	7.00	3	0.10	3
⋮	⋮	⋮	⋮	⋮	⋮	⋮	⋮	⋮
$O_{216}$	0.76	661.50	416.50	122.50	7.00	3	0.10	4
⋮	⋮	⋮	⋮	⋮	⋮	⋮	⋮	⋮
$O_{242}$	0.62	808.50	367.50	220.50	3.50	5	0.10	4
⋮	⋮	⋮	⋮	⋮	⋮	⋮	⋮	⋮
$O_{310}$	0.79	637.00	343.00	147.00	7.00	5	0.25	1
⋮	⋮	⋮	⋮	⋮	⋮	⋮	⋮	⋮
$O_{385}$	0.74	686.00	245.00	220.50	3.50	2	0.25	3

**Table 15:** The eight building shapes.

	$S_{1}$	$S_{2}$	$S_{3}$	$S_{4}$	$S_{5}$
$O_{1}$	0.98	514.50	294.00	110.25	3
$O_{2}$	0.90	563.50	318.50	122.50	5
$O_{3}$	0.76	661.50	416.50	122.50	3
$O_{4}$	0.98	514.50	294.00	110.25	3
$O_{5}$	0.62	808.50	367.50	220.50	4
$O_{6}$	0.82	612.50	318.50	147.00	4
$O_{7}$	0.69	735.00	294.00	220.50	5
$O_{8}$	0.79	637.00	343.00	147.00	2

**Table 16:** The eight building shapes after normalization.

	$S_{1}$	$S_{2}$	$S_{3}$	$S_{4}$	$S_{5}$
$O_{1}$	1.0000	0.6364	1.0000	0.5000	0.6000
$O_{2}$	0.9184	0.6970	0.9231	0.5556	1.0000
$O_{3}$	0.7755	0.8182	0.7059	0.5556	0.6000
$O_{4}$	1.0000	0.6364	1.0000	0.5000	0.6000
$O_{5}$	0.6327	1.0000	0.8000	1.0000	0.8000
$O_{6}$	0.8367	0.7576	0.9231	0.6667	0.8000
$O_{7}$	0.7041	0.9091	1.0000	1.0000	1.0000
$O_{8}$	0.8061	0.7879	0.8571	0.6667	0.4000

**Table 17:** The conditional probability of the eight building shapes.

*O*	$O_{1}$	$O_{2}$	$O_{3}$	$O_{4}$	$O_{5}$	$O_{6}$	$O_{7}$	$O_{8}$
$P(T1|{\left[O_{i}\right]}_{E})$	0.1667	0.5000	0.1429	0.1667	0.5000	0.5000	1.0000	0.1429

**Table 18:** Decision information for the eight building shapes under the TWD ideal superiority model.

*O*	$O_{1}$	$O_{2}$	$O_{3}$	$O_{4}$	$O_{5}$	$O_{6}$	$O_{7}$	$O_{8}$
$\alpha^{E}$	0.5862	0.5075	0.6741	0.5862	0.6243	0.5814	0.3577	0.4673
$\beta^{E}$	0.2912	0.2300	0.3749	0.2912	0.3251	0.2871	0.1390	0.2028
$\gamma^{E}$	0.4327	0.3568	0.5269	0.4327	0.4722	0.4279	0.2307	0.3208
Decision rules	NEG	BND	NEG	NEG	BND	BND	POS	NEG

**Table 19:** The ranking of the eight building shapes under the TWD ideal superiority model.

Domains	Rankings
POS	$O_{7}$
BND	$O_{2}≻O_{5}≻O_{6}$
NEG	$O_{3}≻O_{8}≻O_{1}≻O_{4}$
Overall ranking	$O_{7}≻O_{2}≻O_{5}≻O_{6}≻O_{3}≻O_{8}≻O_{1}≻O_{4}$

**Table 20:** The conditional probabilities for the eight building shapes under the TWD ideal inferiority model.

*O*	$O_{1}$	$O_{2}$	$O_{3}$	$O_{4}$	$O_{5}$	$O_{6}$	$O_{7}$	$O_{8}$
$P(G1|{\left[O_{i}\right]}_{F})$	0.5000	0.6000	0	0.5000	0.6000	0.6000	0.7500	0

**Table 21:** Decision information for the eight building shapes under the TWD ideal inferiority model.

*O*	$O_{1}$	$O_{2}$	$O_{3}$	$O_{4}$	$O_{5}$	$O_{6}$	$O_{7}$	$O_{8}$
$\alpha^{F}$	0.5862	0.5075	0.6741	0.5862	0.6243	0.5814	0.3577	0.4673
$\beta^{F}$	0.2912	0.2300	0.3749	0.2912	0.3251	0.2871	0.1390	0.2028
$\gamma^{F}$	0.4327	0.3568	0.5269	0.4327	0.4722	0.4279	0.2307	0.3208
Decision rules	BND	POS	NEG	BND	BND	POS	POS	NEG

**Table 22:** The ranking of the eight objects under the TWD ideal inferiority model.

Domains	Rankings
POS	$O_{7}≻O_{2}≻O_{6}$
BND	$O_{1}≻O_{4}≻O_{5}$
NEG	$O_{3}≻O_{8}$
Overall ranking	$O_{7}≻O_{2}≻O_{6}≻O_{1}≻O_{4}≻O_{5}≻O_{3}≻O_{8}$

**Table 23:** The ranking results for the eight building shapes in different MADM methods.

Method	Ranking Results	Optimal Object
IS	$O_{7}≻O_{2}≻O_{6}≻O_{5}≻O_{3}≻O_{8}≻O_{1}≻O_{4}$	$O_{7}$
IF	$O_{7}≻O_{2}≻O_{6}≻O_{1}≻O_{4}≻O_{5}≻O_{3}≻O_{8}$	$O_{7}$
TOPSIS	$O_{7}≻O_{2}≻O_{1}≻O_{4}≻O_{6}≻O_{5}≻O_{8}≻O_{3}$	$O_{7}$
PROMETHEE	$O_{7}≻O_{2}≻O_{5}≻O_{6}≻O_{1}≻O_{4}≻O_{8}≻O_{3}$	$O_{7}$
Ye’s	$O_{7}≻O_{5}≻O_{2}≻O_{6}≻O_{1}≻O_{4}≻O_{3}≻O_{8}$	$O_{7}$
Zhang’s	$O_{7}≻O_{2}≻O_{6}≻O_{5}≻O_{1}≻O_{4}≻O_{8}≻O_{3}$	$O_{7}$
Jia’s	$O_{7}≻O_{2}≻O_{5}≻O_{6}≻O_{1}≻O_{4}≻O_{8}≻O_{3}$	$O_{7}$

**Table 24:** The SRCC between any two of the seven decision-making methods.

Method	IS	IF	TOPSIS	PROMETHEE	Ye’s	Zhang’s	Jia’s
IS	1.0000	0.5714	0.3810	0.7857	0.7857	0.7619	0.7857
IF	0.5714	1.0000	0.9048	0.8333	0.7619	0.9048	0.8333
TOPSIS	0.3810	0.9048	1.0000	0.7857	0.6667	0.8095	0.7857
PROMETHEE	0.7857	0.8333	0.7857	1.0000	0.9524	0.9762	1.0000
Ye’s	0.7857	0.7619	0.6667	0.9524	1.0000	0.9048	0.9524
Zhang’s	0.7857	0.9048	0.8095	0.9762	0.9048	1.0000	0.9762
Jia’s	0.7619	0.8333	0.7857	1.0000	0.9524	0.9762	1.0000

**Table 25:** The data of the example in [3].

	$S_{1}$	$S_{2}$	$S_{3}$	$S_{4}$	$S_{5}$
$O_{1}$	0.8	0.4	0.3	0.8	0.9
$O_{2}$	0.9	0.5	0.5	0.7	0.6
$O_{3}$	0.3	0.4	0.6	0.4	0.3
$O_{4}$	0.5	0.2	0.2	0.7	0.6
$O_{5}$	0.7	0.6	0.6	0.5	0.8
$O_{6}$	0.4	0.8	0.7	0.7	0.3
$O_{7}$	0.9	0.5	0.1	0.8	0.7
$O_{8}$	0.6	0.8	0.8	0.3	0.4

**Table 26:** The ranking results for the eight investment objects of different MADM methods.

Method	Ranking Results	Optimal Object
IS	$O_{7}≻O_{2}≻O_{5}≻O_{1}≻O_{4}≻O_{6}≻O_{8}≻O_{3}$	$O_{7}$
IF	$O_{7}≻O_{4}≻O_{2}≻O_{5}≻O_{1}≻O_{3}≻O_{6}≻O_{8}$	$O_{7}$
TOPSIS	$O_{7}≻O_{1}≻O_{2}≻O_{4}≻O_{5}≻O_{6}≻O_{8}≻O_{3}$	$O_{7}$
PROMETHEE	$O_{7}≻O_{1}≻O_{2}≻O_{4}≻O_{5}≻O_{6}≻O_{8}≻O_{3}$	$O_{7}$
Ye’s	$O_{7}≻O_{1}≻O_{4}≻O_{2}≻O_{5}≻O_{6}≻O_{3}≻O_{8}$	$O_{7}$
Zhang’s	$O_{7}≻O_{1}≻O_{4}≻O_{2}≻O_{5}≻O_{6}≻O_{8}≻O_{3}$	$O_{7}$
Jia’s	$O_{7}≻O_{1}≻O_{4}\approx O_{2}≻O_{5}≻O_{6}≻O_{3}≻O_{8}$	$O_{7}$

**Table 27:** The SRCC values between any two of the seven decision-making methods.

Method	IS	IF	TOPSIS	PROMETHEE	Ye’s	Zhang’s	Jia’s
IS	1.0000	0.7857	0.8810	0.8810	0.7857	0.8095	0.7857
IF	0.7857	1.0000	0.7619	0.7619	0.8333	0.7857	0.8333
TOPSIS	0.8810	0.7619	1.0000	1.0000	0.9524	0.9762	0.9524
PROMETHEE	0.8810	0.7619	1.0000	1.0000	0.9524	0.9762	0.9524
Ye’s	0.7857	0.8333	0.9524	0.9524	1.0000	0.9762	1.0000
Zhang’s	0.8095	0.7857	0.9762	0.9762	0.9762	1.0000	0.9762
Jia’s	0.7857	0.8333	0.9524	0.9524	1.0000	0.9762	1.0000

**Table 28:** The data of the example in [16].

	$S_{1}$	$S_{2}$	$S_{3}$	$S_{4}$
$O_{1}$	0.80	0.69	0.64	0.74
$O_{2}$	0.65	0.85	0.72	0.67
$O_{3}$	0.73	0.77	0.78	0.61
$O_{4}$	0.82	0.68	0.64	0.75
$O_{5}$	0.54	0.96	0.57	0.82
$O_{6}$	0.88	0.62	0.70	0.69

**Table 29:** The ranking results for six investment objects for different MADM methods.

Method	Ranking Results	Optimal Object
IS	$O_{6}≻O_{4}≻O_{1}≻O_{2}≻O_{5}≻O_{3}$	$O_{6}$
IF	$O_{6}≻O_{4}≻O_{1}≻O_{5}≻O_{2}≻O_{3}$	$O_{6}$
TOPSIS	$O_{6}≻O_{4}≻O_{1}≻O_{3}≻O_{2}≻O_{5}$	$O_{6}$
PROMETHEE	$O_{6}≻O_{4}≻O_{1}≻O_{3}≻O_{2}≻O_{5}$	$O_{6}$
Zhang’s	$O_{6}≻O_{4}≻O_{1}≻O_{2}≻O_{3}≻O_{5}$	$O_{6}$

**Table 30:** The SRCC between any two of five decision-making methods.

Method	IS	IF	TOPSIS	PROMETHEE	Zhang’s
IS	1.0000	0.9429	0.8286	0.8286	0.9429
IF	0.9429	1.0000	0.7714	0.7714	0.8286
TOPSIS	0.8286	0.7714	1.0000	1.0000	0.9429
PROMETHEE	0.8286	0.7714	1.0000	1.0000	0.9429
Zhang’s	0.9429	0.8286	0.9429	0.9429	1.0000

**Table 31:** The ranking results of different preference parameters $k_{1}$.

Preference Parameter $k_{1}$	Ranking Results
$k_{1}$ = 0.05	$O_{1}≻O_{2}≻O_{3}≻O_{4}≻O_{5}≻O_{6}≻O_{7}≻O_{8}$
$k_{1}$ = 0.10	$O_{1}≻O_{2}≻O_{3}≻O_{4}≻O_{5}≻O_{6}≻O_{7}≻O_{8}$
$k_{1}$ = 0.15	$O_{7}≻O_{2}≻O_{5}≻O_{6}≻O_{3}≻O_{8}≻O_{1}≻O_{4}$
$k_{1}$ = 0.20	$O_{7}≻O_{2}≻O_{5}≻O_{6}≻O_{3}≻O_{8}≻O_{1}≻O_{4}$
$k_{1}$ = 0.25	$O_{2}≻O_{5}≻O_{6}≻O_{7}≻O_{1}≻O_{4}≻O_{8}≻O_{3}$
$k_{1}$ = 0.30	$O_{2}≻O_{5}≻O_{6}≻O_{7}≻O_{1}≻O_{4}≻O_{8}≻O_{3}$
$k_{1}$ = 0.35	$O_{2}≻O_{5}≻O_{6}≻O_{7}≻O_{1}≻O_{4}≻O_{8}≻O_{3}$
$k_{1}$ = 0.40	$O_{2}≻O_{5}≻O_{6}≻O_{7}≻O_{1}≻O_{4}≻O_{8}≻O_{3}$
$k_{1}$ = 0.45	$O_{2}≻O_{5}≻O_{6}≻O_{7}≻O_{1}≻O_{4}≻O_{8}≻O_{3}$
$k_{1}$ = 0.50	$O_{2}≻O_{5}≻O_{6}≻O_{7}≻O_{1}≻O_{4}≻O_{8}≻O_{3}$

**Table 32:** The ranking results for different risk-aversion coefficients τ.

The Risk Aversion Coefficient τ	Ranking Results
τ = 0.0	$O_{7}≻O_{1}≻O_{2}≻O_{3}≻O_{4}≻O_{5}≻O_{6}≻O_{8}$
τ = 0.1	$O_{7}≻O_{1}≻O_{4}≻O_{8}≻O_{3}≻O_{2}≻O_{5}≻O_{6}$
τ = 0.2	$O_{7}≻O_{1}≻O_{4}≻O_{8}≻O_{2}≻O_{5}≻O_{6}≻O_{3}$
τ = 0.3	$O_{7}≻O_{5}≻O_{6}≻O_{2}≻O_{3}≻O_{8}≻O_{1}≻O_{4}$
τ = 0.4	$O_{7}≻O_{2}≻O_{5}≻O_{6}≻O_{3}≻O_{8}≻O_{1}≻O_{4}$
τ = 0.5	$O_{7}≻O_{2}≻O_{5}≻O_{6}≻O_{3}≻O_{8}≻O_{1}≻O_{4}$

**Table 33:** The ranking results for different preference parameters $k_{2}$.

Preference Parameter $k_{2}$	Ranking Results
$k_{2}$ = 0.50	$O_{7}≻O_{2}≻O_{6}≻O_{1}≻O_{4}≻O_{5}≻O_{3}≻O_{8}$
$k_{2}$ = 0.55	$O_{7}≻O_{1}≻O_{3}≻O_{4}≻O_{8}≻O_{6}≻O_{5}≻O_{2}$
$k_{2}$ = 0.60	$O_{7}≻O_{1}≻O_{3}≻O_{4}≻O_{8}≻O_{6}≻O_{5}≻O_{2}$
$k_{2}$ = 0.65	$O_{1}≻O_{2}≻O_{3}≻O_{4}≻O_{5}≻O_{6}≻O_{8}≻O_{7}$
$k_{2}$ = 0.70	$O_{1}≻O_{2}≻O_{3}≻O_{4}≻O_{5}≻O_{6}≻O_{8}≻O_{7}$
$k_{2}$ = 0.75	$O_{1}≻O_{2}≻O_{3}≻O_{4}≻O_{5}≻O_{6}≻O_{8}≻O_{7}$
$k_{2}$ = 0.80	$O_{1}≻O_{2}≻O_{3}≻O_{4}≻O_{5}≻O_{6}≻O_{8}≻O_{7}$
$k_{2}$ = 0.85	$O_{1}≻O_{2}≻O_{3}≻O_{4}≻O_{5}≻O_{6}≻O_{8}≻O_{7}$
$k_{2}$ = 0.90	$O_{1}≻O_{2}≻O_{3}≻O_{4}≻O_{5}≻O_{6}≻O_{8}≻O_{7}$
$k_{2}$ = 0.95	$O_{1}≻O_{2}≻O_{3}≻O_{4}≻O_{5}≻O_{6}≻O_{8}≻O_{7}$
$k_{2}$ = 0.10	$O_{1}≻O_{2}≻O_{3}≻O_{4}≻O_{5}≻O_{6}≻O_{8}≻O_{7}$

**Table 34:** The ranking results for different risk-aversion coefficients τ.

Risk Aversion Coefficient τ	Ranking Results
τ = 0.0	$O_{1}≻O_{2}≻O_{4}≻O_{5}≻O_{6}≻O_{7}≻O_{3}≻O_{8}$
τ = 0.1	$O_{7}≻O_{1}≻O_{4}≻O_{6}≻O_{5}≻O_{2}≻O_{3}≻O_{8}$
τ = 0.2	$O_{7}≻O_{1}≻O_{4}≻O_{6}≻O_{5}≻O_{2}≻O_{3}≻O_{8}$
τ = 0.3	$O_{7}≻O_{2}≻O_{1}≻O_{4}≻O_{6}≻O_{5}≻O_{3}≻O_{8}$
τ = 0.4	$O_{7}≻O_{2}≻O_{5}≻O_{6}≻O_{1}≻O_{4}≻O_{3}≻O_{8}$
τ = 0.5	$O_{7}≻O_{2}≻O_{5}≻O_{6}≻O_{1}≻O_{4}≻O_{3}≻O_{8}$

## Data Availability

Data available in a publicly accessible repository. The data presented in this study are openly available in UCI Machine Learning Repository. (http://archive.ics.uci.edu/ml) (accessed on 21 March 2021).

## Abbreviations

The following abbreviations are used in this manuscript:

MADM	Multi-attribute decision-making
TWD	Three-way decision
